# Supplementary material for: A high-throughput screen for TMPRSS2 expression identifies FDA-approved compounds that can limit SARS-CoV-2 entry
Source: Nat Commun. 2021 Jun 23;12:3907. doi: 10.1038/s41467-021-24156-y (PMC8222394; doi:10.1038/s41467-021-24156-y)
Supplement: Supplementary file 1 — Supplementary Information [file 41467_2021_24156_MOESM1_ESM.pdf]

Supplemental Data

A high-throughput screen for TMPRSS2 expression identifies FDA-approved compounds that can limit SARS-CoV-2 entry

Yanwen Chen[1,2*], Travis B. Lear[1,3,4*], John W. Evankovich[1,3*], Mads B. Larsen[1], Bo Lin[1], Irene Alfaras[1], Jason R. Kennerdell[1], Laura Salminen[1], Daniel P. Camarco[1], Karina C. Lockwood[1], Ferhan Tuncer[1], Jie Liu[1], Michael M. Myerburg[3], John F. McDyer[3], Yuan Liu[1,3,5#], Toren Finkel[1,4,6#], and Bill B. Chen[1,3,4,#]

[1] Aging Institute, University of Pittsburgh/UPMC, Pittsburgh, PA 15219, USA
[2] Department of Gastroenterology, Ruijin Hospital, Shanghai Jiaotong University School of Medicine, Shanghai 200025, China
[3] Department of Medicine, Acute Lung Injury Center of Excellence, University of Pittsburgh, Pittsburgh, PA 15213, USA
[4] Vascular Medicine Institute, University of Pittsburgh, Pittsburgh, PA, 15213, USA
[5] McGowan Institute for Regenerative Medicine, University of Pittsburgh, Pittsburgh, PA 15219, USA
[6] Department of Medicine, Division of Cardiology, University of Pittsburgh, Pittsburgh, PA 15213, USA

*These authors contributed equally to this work

Address Correspondence to:
Bill B. Chen, PhD #
Toren Finkel, MD., PhD #
Yuan Liu, PhD #
University of Pittsburgh
Aging Institute, Department of Medicine
558 Bridgeside Point 1
100 Technology Drive
Pittsburgh, PA 15219
E-mail: chenb@upmc.edu, finkelt@pitt.edu, liuy13@upmc.edu

Supplemental Figure Legends:

Supplementary Figure 1: **Assessment of TMPRSS2 HTS Screen**. (**A**) TMPRSS2-HiBiT expressing BEAS-2B cells were seeded into 384-well plates for 24h. Half of the cells were treated with cycloheximide (CHX) overnight and the lytic HiBiT signal was measured. A subsequent Z'-factor was calculated. (**B**) HTS results from the non-lytic extracellular HiBiT detection of TMPRSS2-HiBiT. The top 20 compounds are specified. (**C**) Lytic HiBiT detection reflecting total cellular TMPRSS2-HiBiT; top 20 compound hits are listed. The vehicle datapoint is shown for both screens.

Supplementary Figure 2: IC50 calculations for compounds that reduce TMPRSS2 expression. Selected compounds (top 24 in total) inhibiting TMPRSS2 expression were tested for their potency in reducing extracellular (first column) or total (middle column) TMPRSS2-HiBiT protein levels in TMPRSS2-HiBiT-BEAS-2B cells. An assessment of cellular toxicity for each compound (CellTiter-Glo) is shown in the last column. Data are mean -/+ SEM (n=4 biologically independent samples). Estimated values of IC50 and CC50 (cytotoxicity concentration 50%) are shown.

Supplementary Figure 3: **HHT and HFG decrease TMPRSS2 protein without decreasing gene expression** (**A**)**.** Agents identified in the screen selectively decrease TMPRSS2 in airway cells. TMPRSS2-HiBiT expressing MLE-12 cells were treated with three agents identified in the screen (homoharringtonine, halofuginione, or venetoclax) at the indicated concentration for 18 hr prior to chemiluminescent detection of TMPRSS2-HiBiT expression. Conventional detection of actin is shown as a loading control. (**B**)**.** Analysis of TMPRSS2 mRNA expression by qPCR analysis of Caco-2 cells treated with homoharringtonine or halofuginone for 6 hr. Expression of other related proteases including hepsin (HPN), ST14, and corin were also measured. Data represent mean -/+ SEM (n=4 biologically independent samples). P-values are shown for comparisons to vehicle, or as indicated by one-way ANOVA with Dunnett's test of multiple comparisons.

Supplementary Figure 4: HHT and HFG reduce TMPRSS2 in Calu-3 and Caco-2 cell line. (**A-B**) Calu-3 cells were treated with a dose course of homoharringtonine (HHT) or halofuginone (HFG) for 18 hr prior to immunoblotting analysis. Densitometry of endogenous TMPRSS2 is shown below. Data represent mean -/+ SEM (n=3 biologically independent samples). (**C**) Time-course treatment of HHT-treated Caco-2 cells (3 μM). TMPRSS2 densitometry is shown, data are mean +/- SEM (n=3 biologically independent samples). (**D**) Immunoblot analysis of Caco-2 cells treated with HHT for 18hr prior to removing the drug, adding fresh media, and then analyzing the protein recovery time course. P-values are shown for comparisons to 0 time point or control, or as indicated by one-way ANOVA with Dunnett's test of multiple comparisons (A-D).

Supplementary Figure 5: HHT and HFG do not decrease Ace2 and E-cadherin. (A) Immunoblotting of Caco-2 cells treated with HFG or HHT dose course to measure e-cadherin as a

control membrane protein. (**B**) Ace2-GFP and TMPRSS2-V5 were transfected to Beas-2B cells prior to HFG and HHT treatment. (**C**) Caco-2 cells co-treated with HFG (3 μM) and MLN4924 (MLN, 1μM) or carfilzomib (CFZ, 1μM).

Supplementary Figure 6: Exogenous proline addition ablates HFG effect on TMPRSS2. (**A)** in vitro transcription/translation assay. V5- or HiBiT-tagged TMPRSS2 was treated with HFG without or with L-proline supplementation (n=3 biologically independent samples). **(B)** HFG-induced decrease in TMPRSS-HiBiT signal is blunted with exogenous proline addition to Beas-2B cells. Data represent mean +/- SEM (n=8 biologically independent samples). P-values are shown for comparisons to vehicle or control, or as indicated by one-way ANOVA with Dunnett's test (B)

Supplementary Figure 7: HHT and HFG prevent CoV-2 Pseudoviral entry. (**A-B**) SARS-CoV-2 pseudovirus entry assay in Calu-3 cells following HHT or HFG treatment, corrected for cell viability with Celltiter-Glo and normalized to vehicle treatment. Data are mean -/+ SEM (n=3 biologically independent samples). (**C**) SARS-CoV-2 pseudovirus entry assay in Caco-2 cells treated with calcium channel blocker cilnidipine. Data are mean -/+ SEM (n=3 biologically independent samples). (**D**) Treatment of Caco-2 cells with the indicated agents. All agents were assessed at 10 μM except for camostat (3 μM). Data are mean -/+ SEM (n=3 biologically independent samples), SARS-CoV-2 pseudoviral luminescence is corrected for cell viability with CellTiter-Glo and normalized to vehicle treatment. **(E)** SARS-CoV-2 pseudoviral entry to Caco-2 was assayed with combination of HHT and HFG treatment. Data represent mean -/+ SEM (n=3 biologically independent samples). P-values are shown for comparisons to vehicle or control, or as indicated by one-way ANOVA with Dunnett's test (A-E).

Supplementary Figure 8: HHT and HFG prevent viral entry of FLUC-based CoV-2 pseudoviral system (A-B) Effects of homoharringtonine (A) or halofuginone (B) on pseudoviral-mediated FLUC expression in Caco2 cells. Data are mean -/+ SEM (n=4 biologically independent samples)., SARS-CoV-2 pseudoviral FLUC is corrected for cellular protein concentration and normalized to vehicle treatment. P-values are shown for comparisons to vehicle or control, or as indicated by one-way ANOVA with Dunnett's test (A,B).

Supplementary Figure 9: HHT and HFG do not inhibit TMPRSS2 activity. in vitro fluorescent activity assay of recombinant TMPRSS2 in the presence of a dose course of several compounds. Data are mean -/+ SEM (n=4 biologically independent samples). P-values are shown for comparisons to vehicle or control, or as indicated by two-way ANOVA with Tukey's test (A,B).

Supplementary Figure 10: HFG does not bind TMPRSS2 nor DCAF1, and HHT and HFG do not affect DCAF1 gene expression. Cellular thermal shift assay of TMPRSS2 (**A**) and DCAF1 (**B**) proteins in Beas-2B cells transfected with TMPRSS2-HiBiT plasmid, upon treatment with vehicle or HFG. The abundance of TMPRSS2 and DCAF1 were detected by HiBiT blotting and

anti-DCAF1 antibody respectively. The corresponding quantifications of densitometry were shown beneath. Data are mean -/+ SEM (n=3 biologically independent samples). (**C**) qPCR analysis of DCAF1 gene expression in Caco-2 treated with HFG or HHT (3μM). Data represent mean -/+ SEM (n=3-4 biologically independent samples). P-values are shown for comparisons to vehicle or control, or as indicated by one-way ANOVA with Dunnett's test (A)

Supplementary Figure 11: **Functional impact of Proline and DCAF1 on HFG efficacy.** (**A-B**) SARS-CoV-2 HiBiT-Spike pseudoviral entry assay with Caco-2 (**A**) or Calu-3 (**B**) cells treated with HFG without and with L-proline supplementation. Data represent mean +/- SEM (n=6 biologically independent samples). (**C**) Beas-2B were treated with control or DCAF1 siRNA prior to HFG-treatment and Cov-2 pseudoviral entry assay. Data represent mean -/+ SEM (n=6 biologically independent samples). P-values are shown for comparisons to vehicle or control, or as indicated by one-way ANOVA with Tukey's test (A-B) of multiple comparisons or by two-sided unpaired t-test (C).

Supplementary Figure 12: Strategies to limit SARS-CoV-2 infection. Agents identified here act by reducing the expression of TMPRSS2 and thereby limiting viral entry. Combinations of these agents may synergize which each other to reduce TMPRSS2 levels below what a single agent can achieve. In addition, agents that reduce TMPRSS2 levels or function would also likely synergize with agents (e.g. chloroquine) that predominantly target viral entry through the endosomal pathway.

Supplementary Table 1: Oligonucleotide Sequences

Primer name	Primer sequence
hTMPRSS2_HiBiT	AGGAGCGATCGCCATGGCTTTGAACTCAGGGTCAC
	CTTGGTTTAAAC GCCGTCTGCCCTCATTTGTC
hTMPRSS2_pcDNA3.1D	CACC ATGGCTTTGAACTCAGGGTC
	GCCGTCTGCCCTCATTTGTC
hTMPRSS2_K70R	GCACGCAGCCCAGATCCCCATCCGG
	CCGGATGGGGATCTGGGCTGCGTGC
hTMPRSS2_K80R	CAGTGTGCACCTCAAGGACTAAGAAAGCAC
	GTGCTTTCTTAGTCCTTGAGGTGCACACTG
hTMPRSS2_KK82RR	GTGCACCTCAAAGACTAGGCGAGCACTGTGCATCACC
	GGTGATGCACAGTGCTCGCCTAGTCTTTGAGGTGCAC
SARS-CoV-2_Spike_HiBiT	CACCGCGATCGCCATGTTTGTGTTCCTGGTGCTGC
	GTCGGTTTAAACACAACAGGAGCCACAGGAACA
hTMPRSS2_qPCR1	CTGACTTTCAACGACCTAGTG
	TTCTGAGGTCTTCCCTTTCT
hTMPRSS2_qPCR2	GGGACATGGGCTATAAGAATAA
	CAGGCTATACAGCGTAAAGAA
hTMPRSS2_qPCR3	CCTCTAACTGGTGTGATGGCGT
	TGCCAGGACTTCCTCTGAGATG
hDCAF1_qPCR	TTGCATACTGTTCCCGCTC
	AATGTTCGGAAGGATGACCC
hHepsin_qPCR	GCCATTGTGGCTGTTCT
	CCCTTCCGTCTTGTCAAAG
hST14_qPCR	AGTCAACAACGTCAAGAAGG
	GTCCCGGTACTGCAAATG
hCorin_qPCR	CCAAGGGTCCGATGTTATTC
	CATACAGGCACTTGTATTCCT

Fig. S1

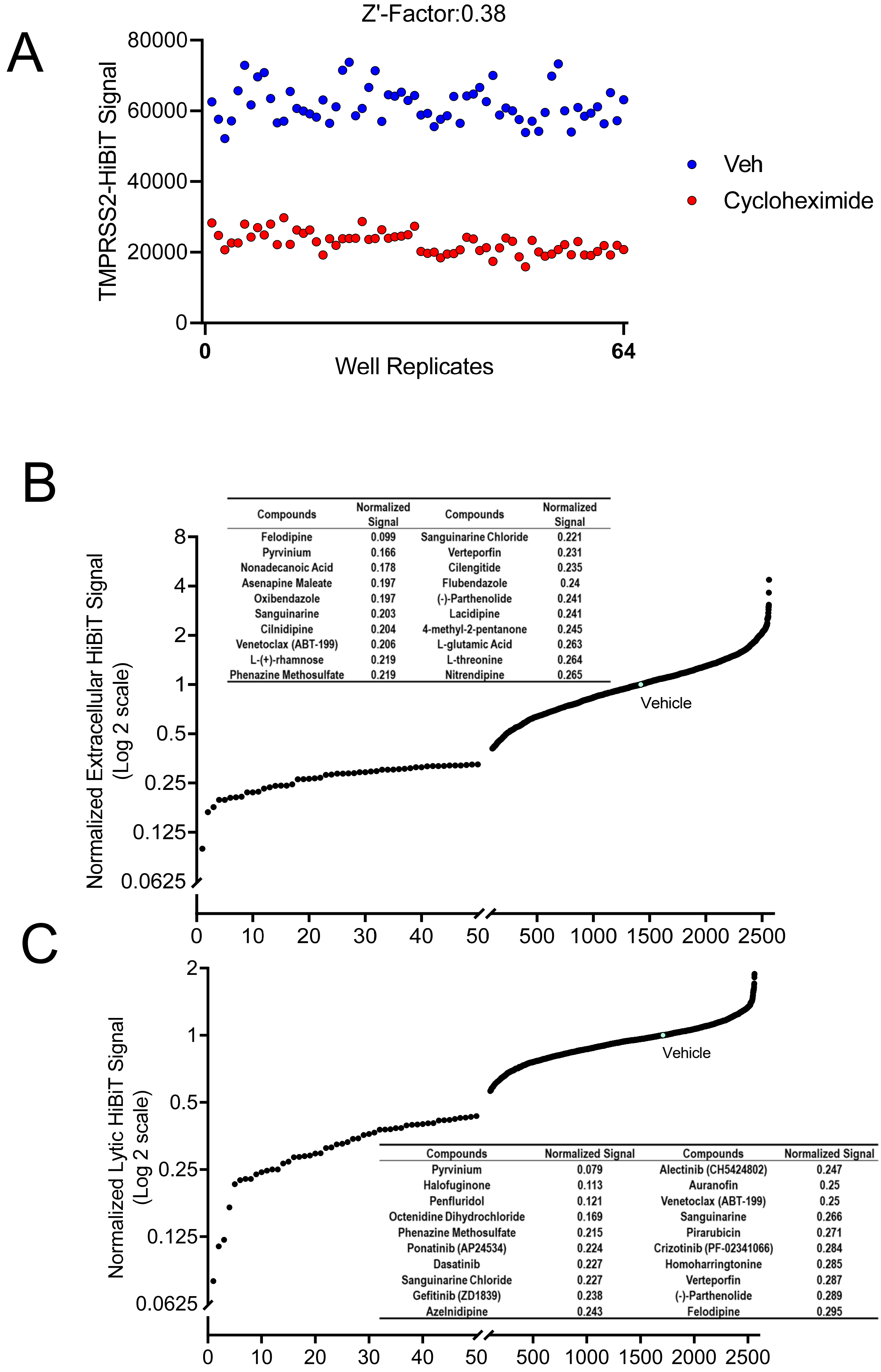

Compounds	Normalized Signal	Compounds	Normalized Signal
Felodipine	0.099	Sanguinarine Chloride	0.221
Pyrvinium	0.166	Verteporfin	0.231
Nonadecanoic Acid	0.178	Cilengitide	0.235
Asenapine Maleate	0.197	Flubendazole	0.24
Oxibendazole	0.197	(-)-Parthenolide	0.241
Sanguinarine	0.203	Lacidipine	0.241
Cilnidipine	0.204	4-methyl-2-pentanone	0.245
Venetoclax (ABT-199)	0.206	L-glutamic Acid	0.263
L-(+)-rhamnose	0.219	L-threonine	0.264
Phenazine Methosulfate	0.219	Nitrendipine	0.265

Compounds	Normalized Signal	Compounds	Normalized Signal
Pyrvinium	0.079	Alectinib (CH5424802)	0.247
Halofuginone	0.113	Auranofin	0.25
Penfluridol	0.121	Venetoclax (ABT-199)	0.25
Octenidine Dihydrochloride	0.169	Sanguinarine	0.266
Phenazine Methosulfate	0.215	Pirarubicin	0.271
Ponatinib (AP24534)	0.224	Crizotinib (PF-02341066)	0.284
Dasatinib	0.227	Homoharringtonine	0.285
Sanguinarine Chloride	0.227	Verteporfin	0.287
Gefitinib (ZD1839)	0.238	(-)-Parthenolide	0.289
Azelnidipine	0.243	Felodipine	0.295

Fig. S2

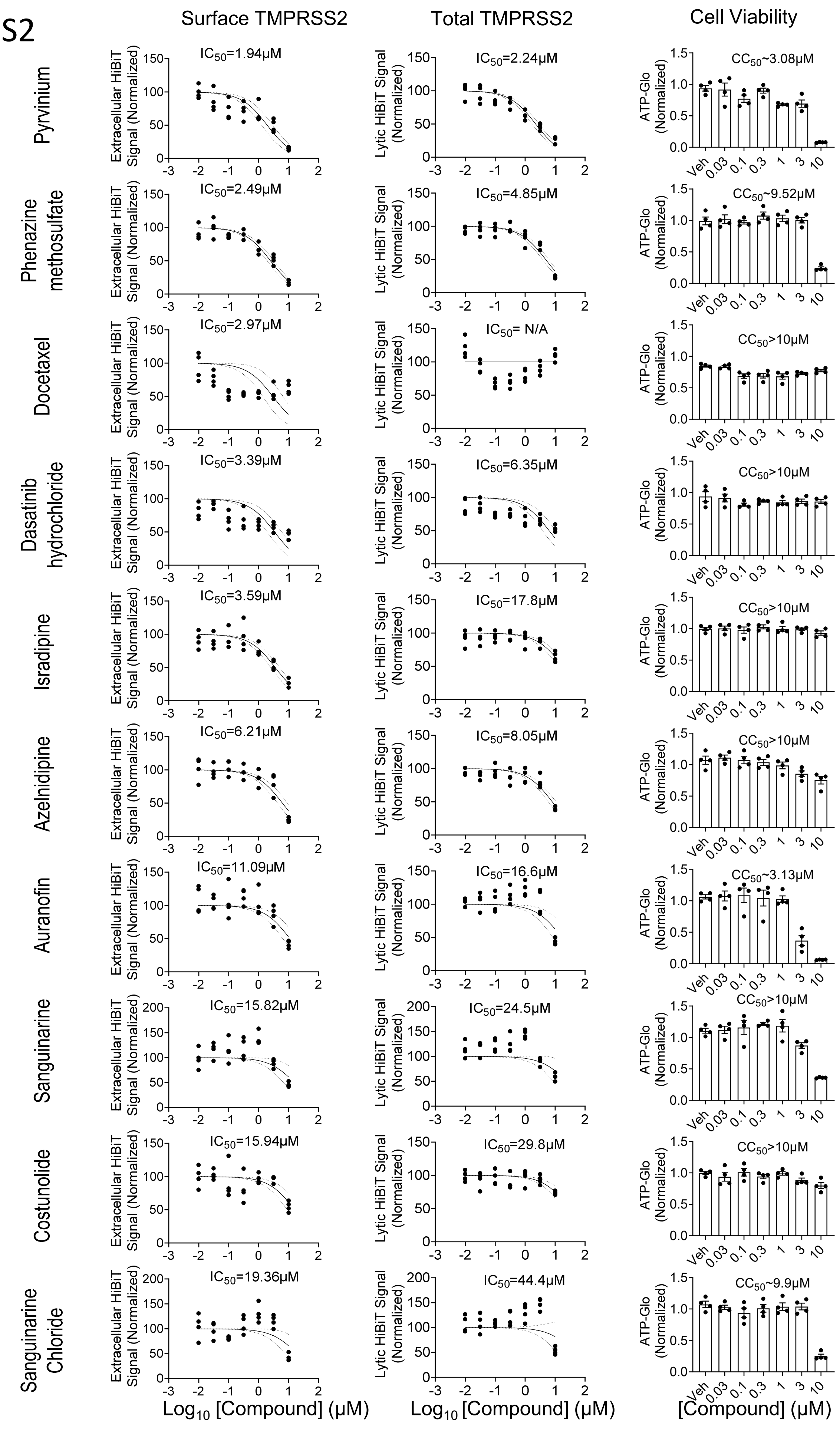

Surface TMPRSS2
Total TMPRSS2
Cell Viability
Pyrvinium
IC_{50}=1.94μM
IC_{50}=2.24μM
CC_{50}~3.08μM
Phenazine methosulfate
IC_{50}=2.49μM
IC_{50}=4.85μM
CC_{50}~9.52μM
Docetaxel
IC_{50}=2.97μM
IC_{50}= N/A
CC_{50}>10μM
Dasatinib hydrochloride
IC_{50}=3.39μM
IC_{50}=6.35μM
CC_{50}>10μM
Isradipine
IC_{50}=3.59μM
IC_{50}=17.8μM
CC_{50}>10μM
Azelnidipine
IC_{50}=6.21μM
IC_{50}=8.05μM
CC_{50}>10μM
Auranofin
IC_{50}=11.09μM
IC_{50}=16.6μM
CC_{50}~3.13μM
Sanguinarine
IC_{50}=15.82μM
IC_{50}=24.5μM
CC_{50}>10μM
Costunolide
IC_{50}=15.94μM
IC_{50}=29.8μM
CC_{50}>10μM
Sanguinarine Chloride
IC_{50}=19.36μM
IC_{50}=44.4μM
CC_{50}~9.9μM
Extracellular HiBiT Signal (Normalized)
Lytic HiBiT Signal (Normalized)
ATP-Glo (Normalized)
Veh 0.03 0.1 0.3 1 3 10
Log_{10} [Compound] (μM)
Log_{10} [Compound] (μM)
[Compound] (μM)

Fig. S2 (cont)

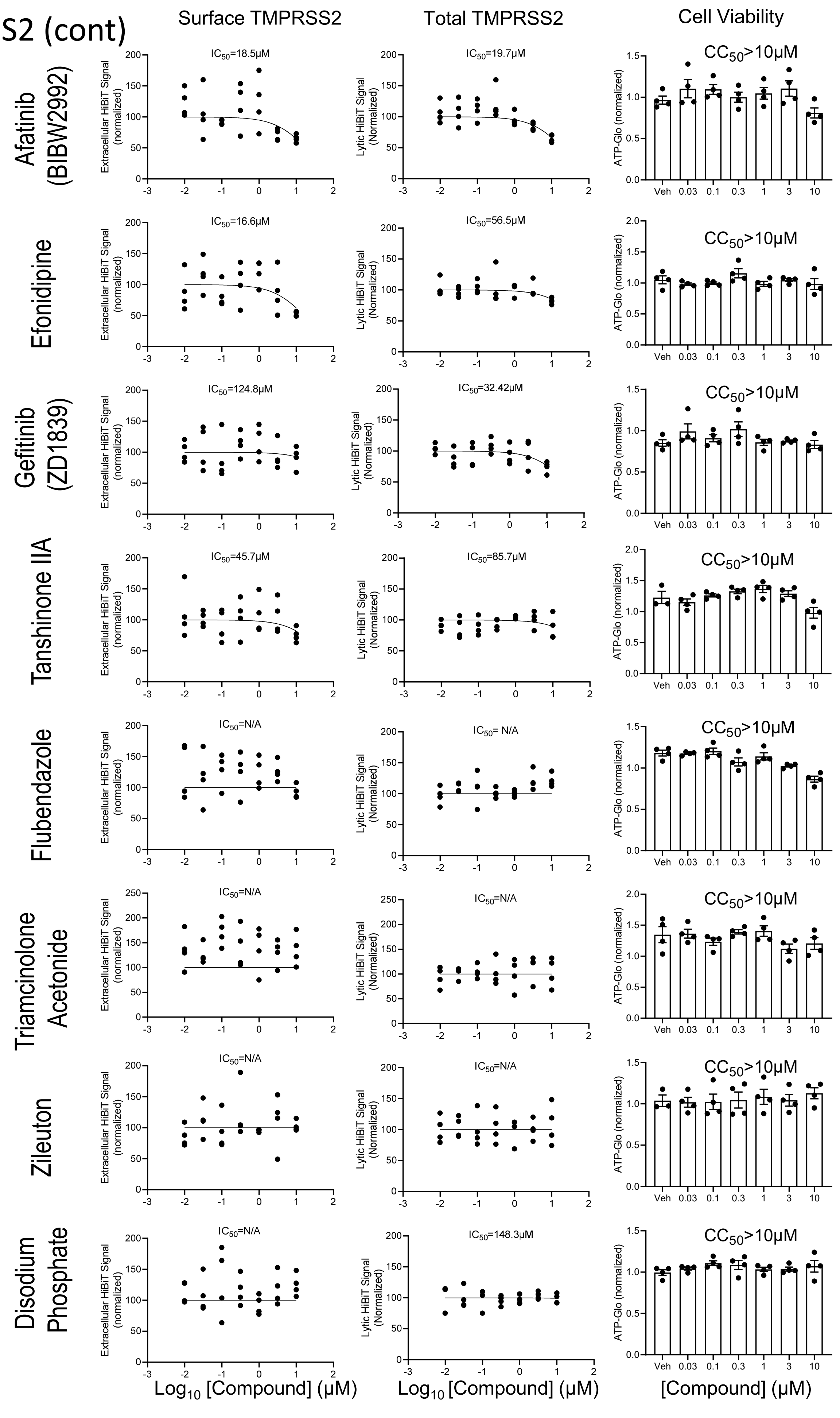

Surface TMPRSS2
Total TMPRSS2
Cell Viability
Afatinib (BIBW2992)
IC50=18.5μM
IC50=19.7μM
CC50>10μM
Efonidipine
IC50=16.6μM
IC50=56.5μM
CC50>10μM
Gefitinib (ZD1839)
IC50=124.8μM
IC50=32.42μM
CC50>10μM
Tanshinone IIA
IC50=45.7μM
IC50=85.7μM
CC50>10μM
Flubendazole
IC50=N/A
IC50= N/A
CC50>10μM
Triamcinolone Acetonide
IC50=N/A
IC50=N/A
CC50>10μM
Zileuton
IC50=N/A
IC50=N/A
CC50>10μM
Disodium Phosphate
IC50=N/A
IC50=148.3μM
CC50>10μM
Extracellular HiBiT Signal (normalized)
Lytic HiBiT Signal (Normalized)
ATP-Glo (normalized)
Veh
0.03
0.1
0.3
1
3
10
Log10 [Compound] (μM)
Log10 [Compound] (μM)
[Compound] (μM)

Fig. S3

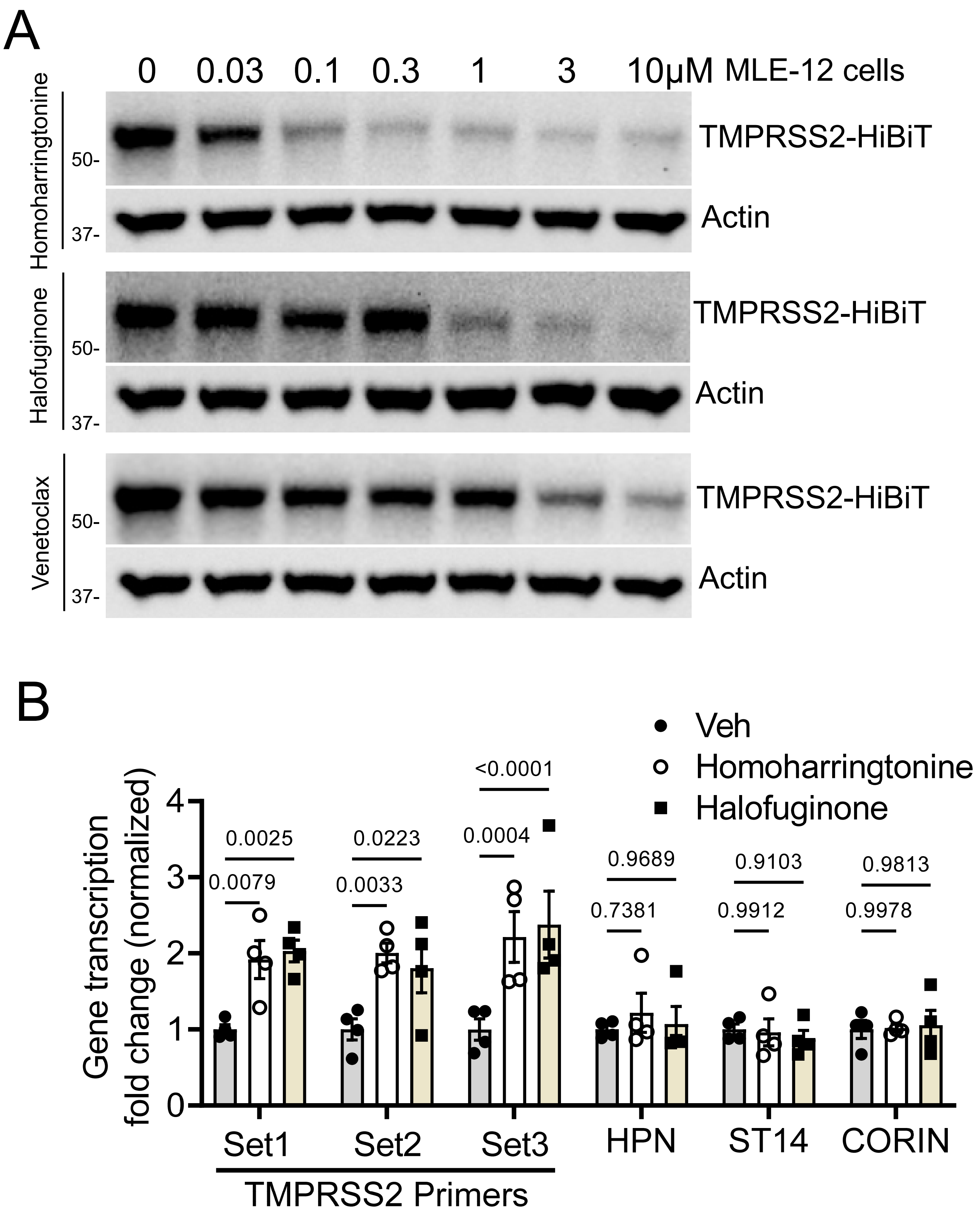
A
0
0.03
0.1
0.3
1
3
10μM
MLE-12 cells
Homoharringtonine
Halofuginone
Venetoclax
TMPRSS2-HiBiT
Actin
50-
37-
B
Veh
Homoharringtonine
Halofuginone
Gene transcription
fold change (normalized)
0
1
2
3
4
0.0025
0.0079
0.0223
0.0033
<0.0001
0.0004
0.9689
0.7381
0.9103
0.9912
0.9813
0.9978
Set1
Set2
Set3
HPN
ST14
CORIN
TMPRSS2 Primers

Fig. S4

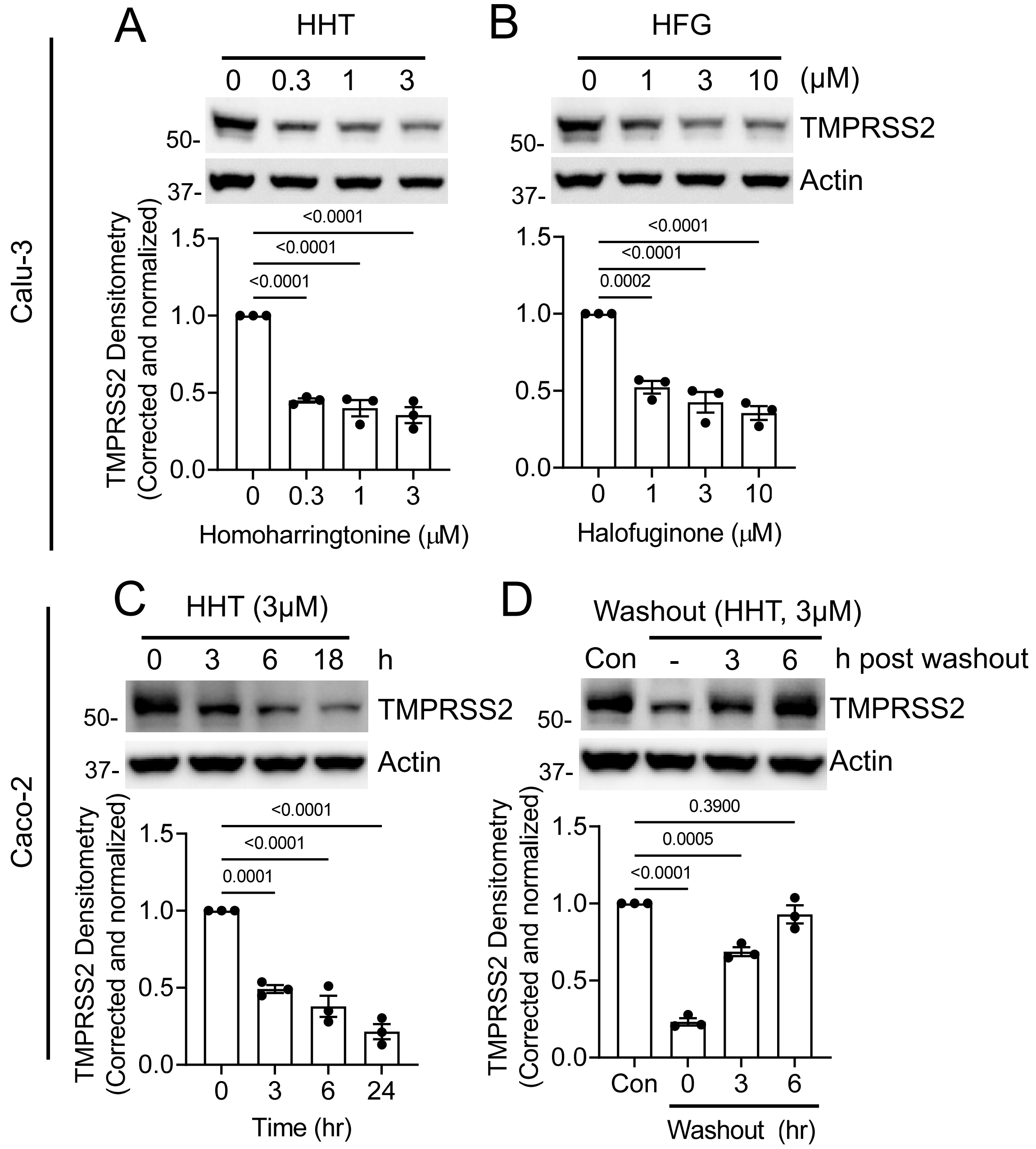

Calu-3
A
HHT
0 0.3 1 3
50-
37-
<0.0001
<0.0001
<0.0001
TMPRSS2 Densitometry (Corrected and normalized)
1.5
1.0
0.5
0.0
0 0.3 1 3
Homoharringtonine (μM)
B
HFG
0 1 3 10 (μM)
TMPRSS2
Actin
50-
37-
<0.0001
<0.0001
0.0002
1.5
1.0
0.5
0.0
0 1 3 10
Halofuginone (μM)
Caco-2
C
HHT (3μM)
0 3 6 18 h
TMPRSS2
Actin
50-
37-
<0.0001
<0.0001
0.0001
TMPRSS2 Densitometry (Corrected and normalized)
1.5
1.0
0.5
0.0
0 3 6 24
Time (hr)
D
Washout (HHT, 3μM)
Con - 3 6 h post washout
TMPRSS2
Actin
50-
37-
0.3900
0.0005
<0.0001
TMPRSS2 Densitometry (Corrected and normalized)
1.5
1.0
0.5
0.0
Con 0 3 6
Washout (hr)

Fig. S5

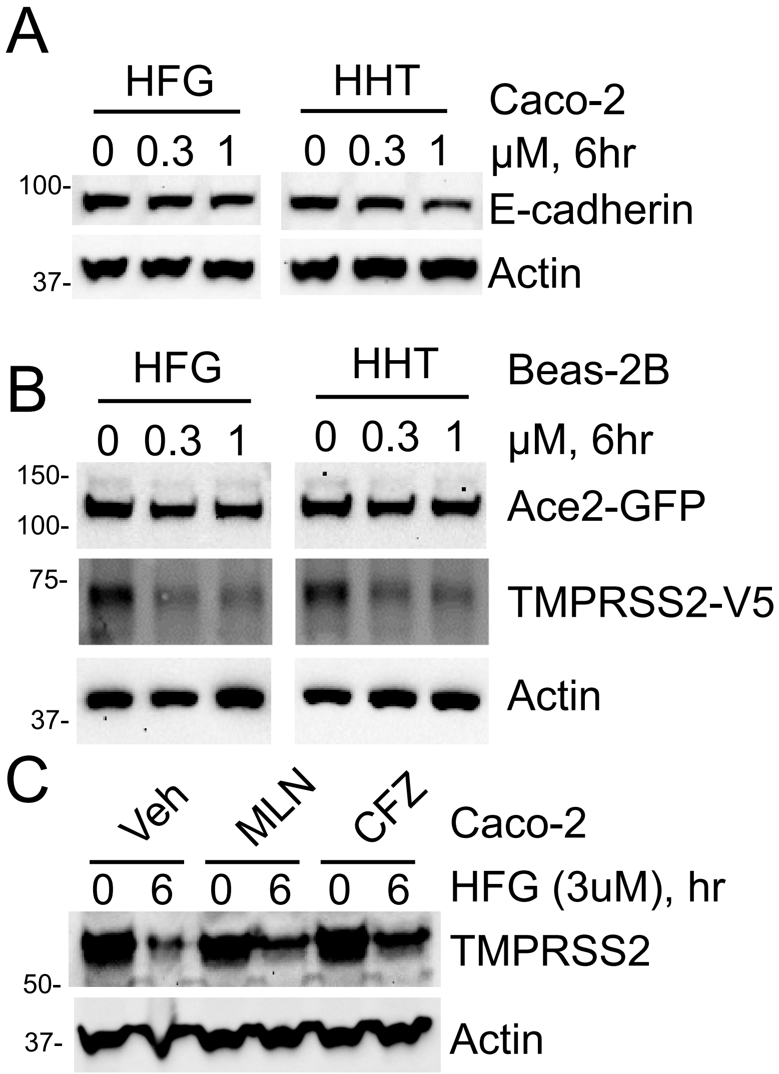
A
HFG
HHT
Caco-2
0 0.3 1 0 0.3 1
µM, 6hr
100-
E-cadherin
37-
Actin
B
HFG
HHT
Beas-2B
0 0.3 1 0 0.3 1
µM, 6hr
150-
100-
Ace2-GFP
75-
TMPRSS2-V5
37-
Actin
C
Veh
MLN
CFZ
Caco-2
0 6 0 6 0 6
HFG (3uM), hr
TMPRSS2
50-
37-
Actin

Fig. S6

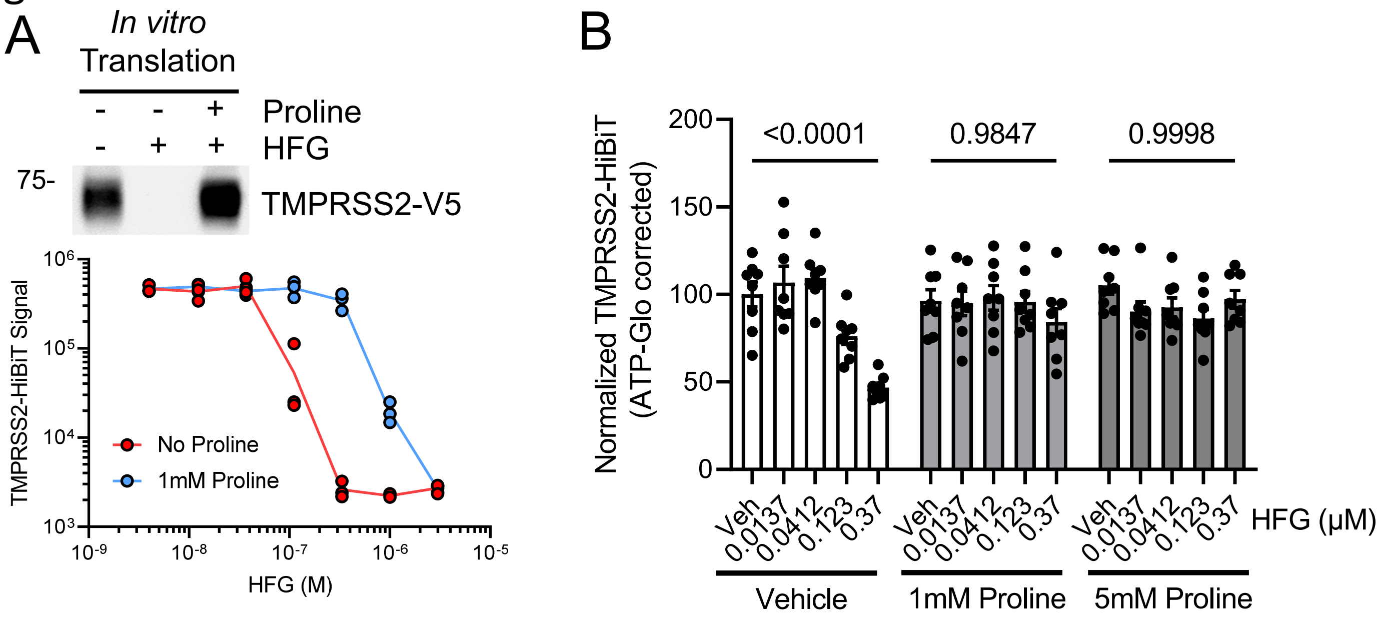
A
In vitro Translation
Proline
HFG
75-
TMPRSS2-V5
TMPRSS2-HiBiT Signal
No Proline
1mM Proline
HFG (M)
B
Normalized TMPRSS2-HiBiT (ATP-Glo corrected)
<0.0001
0.9847
0.9998
Veh
0.0137
0.0412
0.123
0.37
HFG (μM)
Vehicle
1mM Proline
5mM Proline

Fig. S7

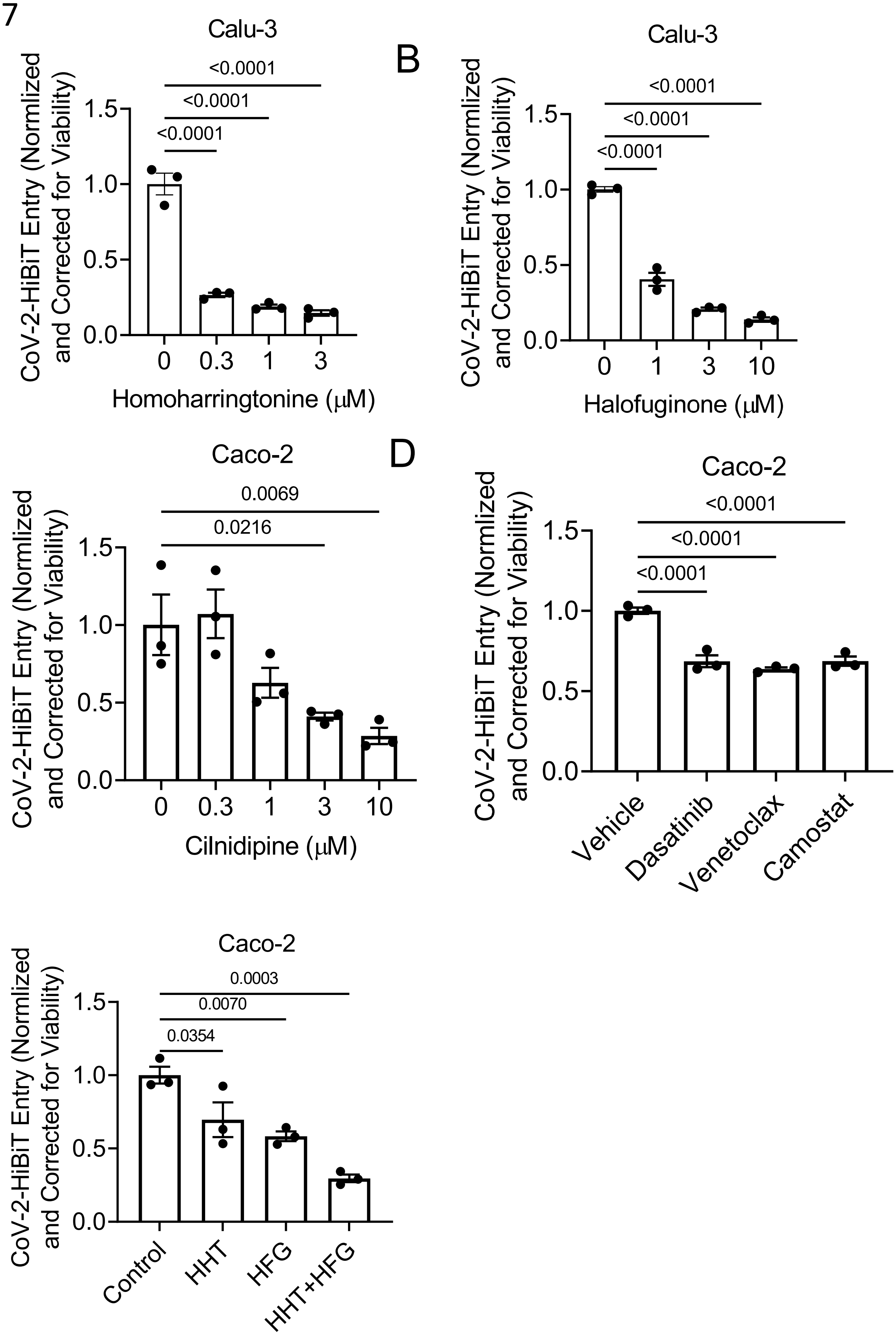

A
Calu-3
CoV-2-HiBiT Entry (Normlized and Corrected for Viability)
<0.0001
<0.0001
<0.0001
1.5
1.0
0.5
0.0
0
0.3
1
3
Homoharringtonine (μM)
B
Calu-3
CoV-2-HiBiT Entry (Normlized and Corrected for Viability)
<0.0001
<0.0001
<0.0001
1.5
1.0
0.5
0.0
0
1
3
10
Halofuginone (μM)
C
Caco-2
CoV-2-HiBiT Entry (Normlized and Corrected for Viability)
0.0069
0.0216
1.5
1.0
0.5
0.0
0
0.3
1
3
10
Cilnidipine (μM)
D
Caco-2
CoV-2-HiBiT Entry (Normlized and Corrected for Viability)
<0.0001
<0.0001
<0.0001
1.5
1.0
0.5
0.0
Vehicle
Dasatinib
Venetoclax
Camostat
E
Caco-2
CoV-2-HiBiT Entry (Normlized and Corrected for Viability)
0.0003
0.0070
0.0354
1.5
1.0
0.5
0.0
Control
HHT
HFG
HHT+HFG

Fig. S8

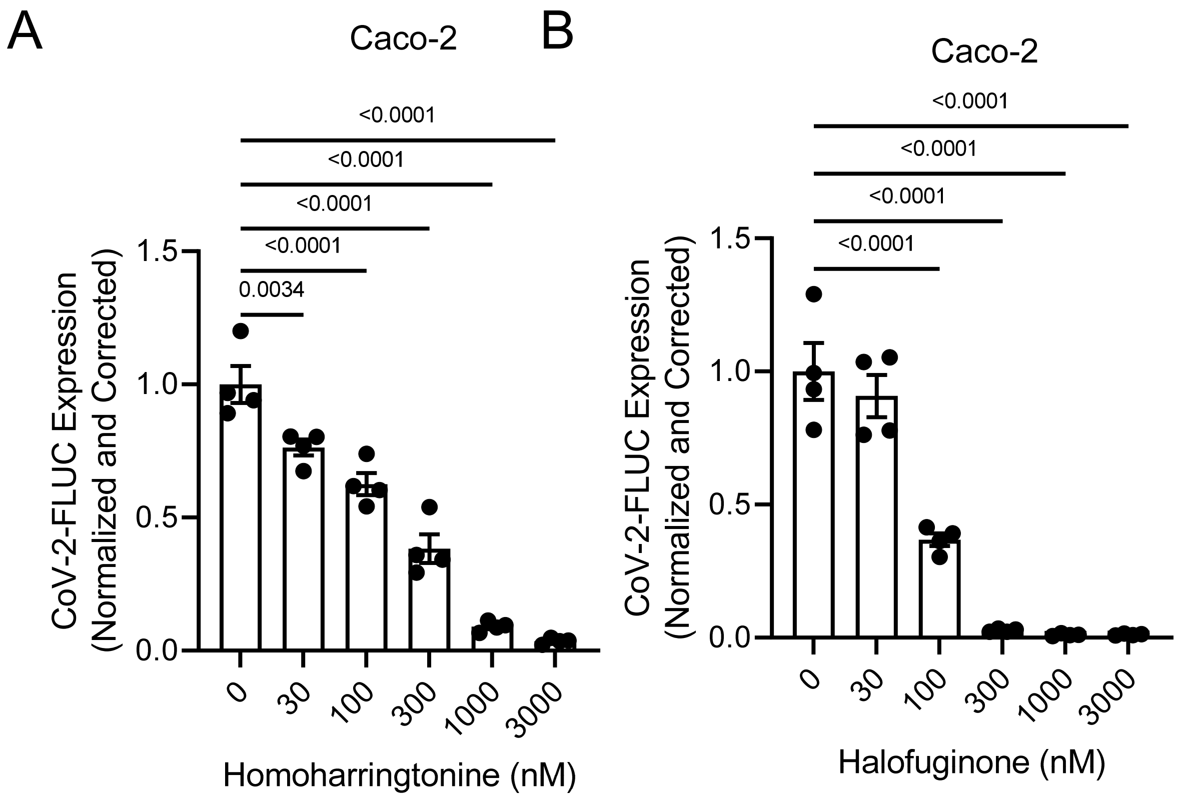
A
Caco-2
<0.0001
<0.0001
<0.0001
<0.0001
0.0034
CoV-2-FLUC Expression (Normalized and Corrected)
1.5
1.0
0.5
0.0
0
30
100
300
1000
3000
Homoharringtonine (nM)
B
Caco-2
<0.0001
<0.0001
<0.0001
<0.0001
CoV-2-FLUC Expression (Normalized and Corrected)
1.5
1.0
0.5
0.0
0
30
100
300
1000
3000
Halofuginone (nM)

Fig. S9

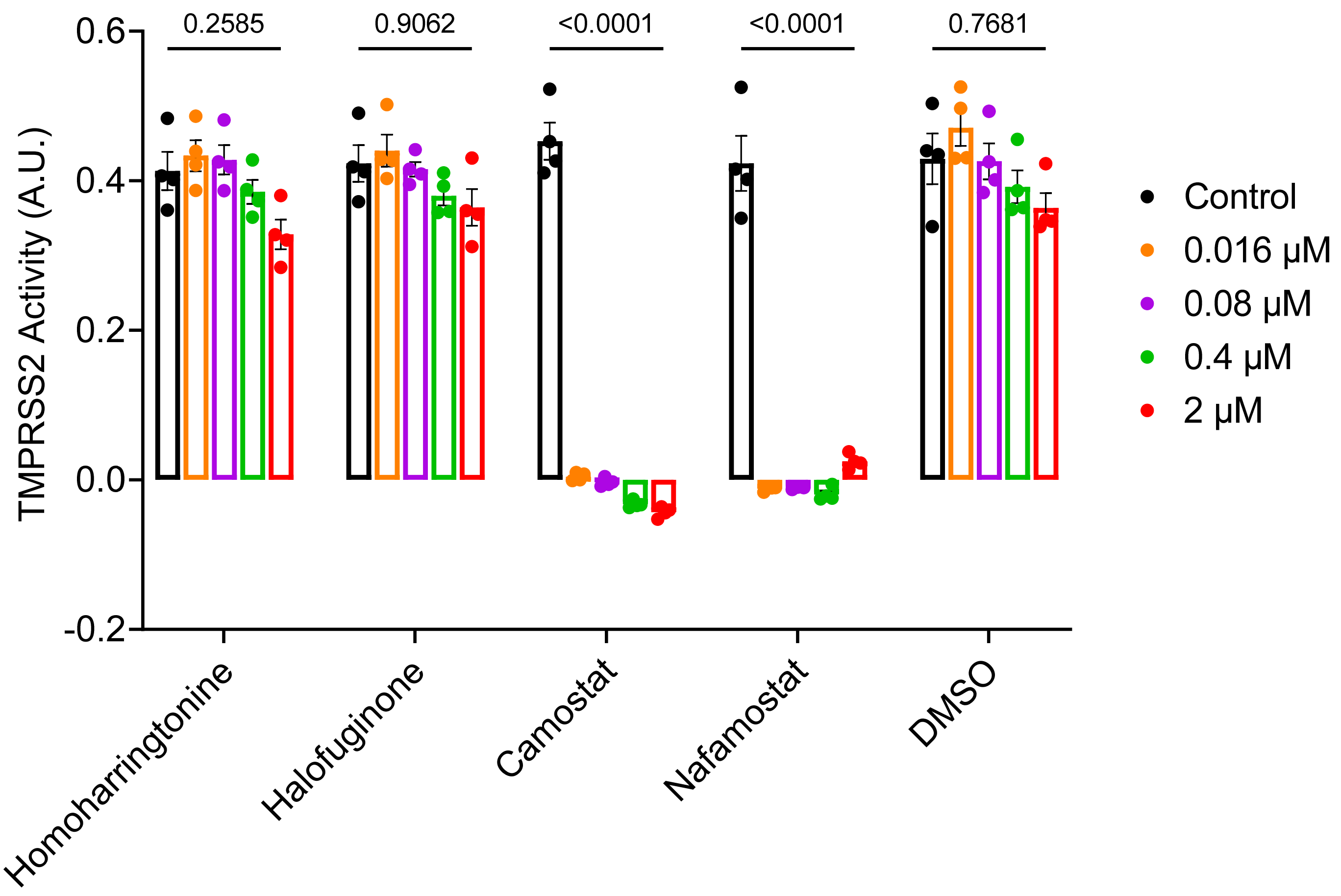
0.2585
0.9062
<0.0001
<0.0001
0.7681
TMPRSS2 Activity (A.U.)
0.6
0.4
0.2
0.0
-0.2
Homoharringtonine
Halofuginone
Camostat
Nafamostat
DMSO
Control
0.016 μM
0.08 μM
0.4 μM
2 μM

Fig. S10

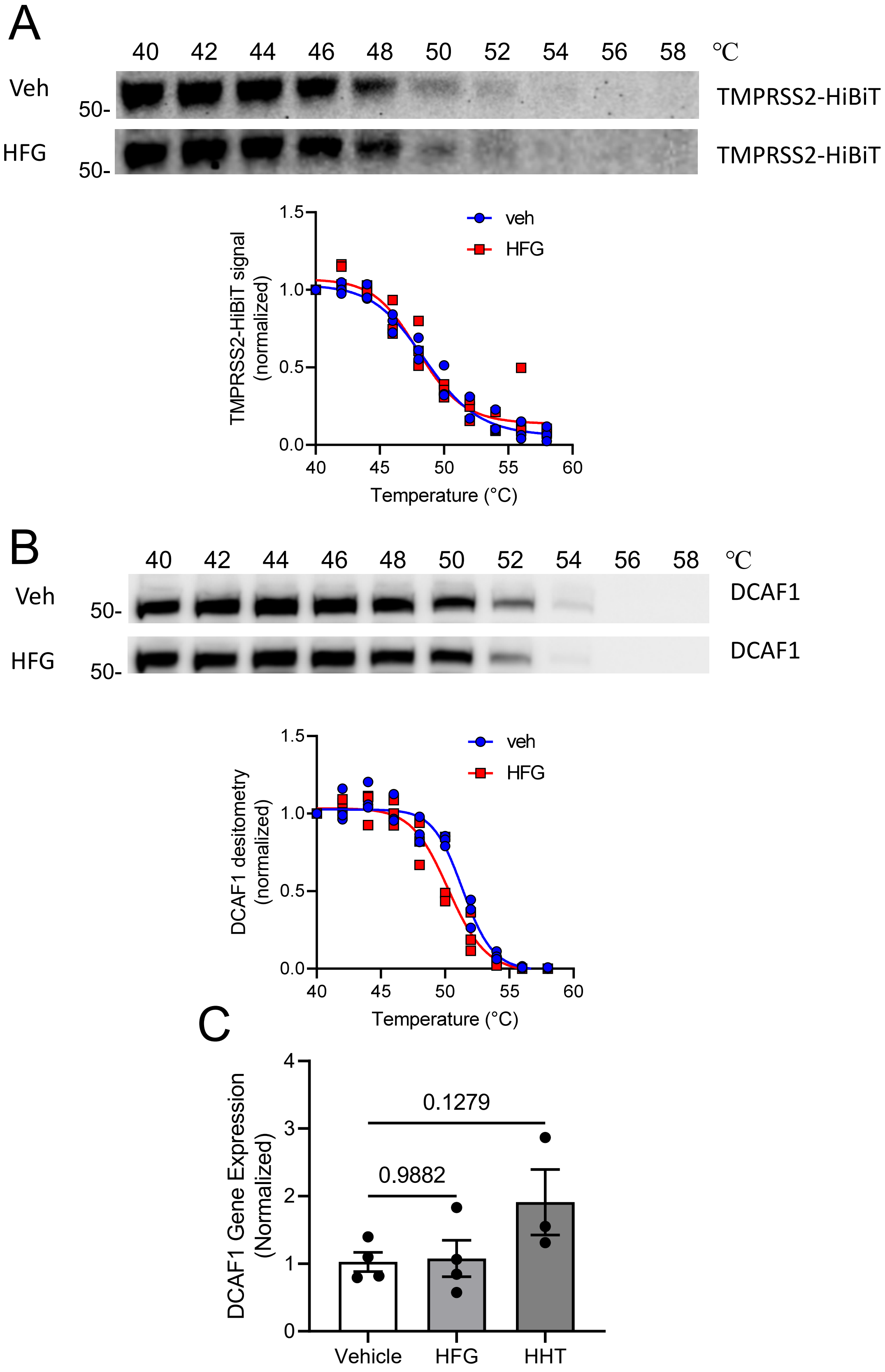

A
40 42 44 46 48 50 52 54 56 58 ℃
Veh
50-
TMPRSS2-HiBiT
HFG
50-
TMPRSS2-HiBiT
TMPRSS2-HiBiT signal (normalized)
veh
HFG
Temperature (°C)
B
40 42 44 46 48 50 52 54 56 58 ℃
Veh
50-
DCAF1
HFG
50-
DCAF1
DCAF1 desitometry (normalized)
veh
HFG
Temperature (°C)
C
DCAF1 Gene Expression (Normalized)
0.1279
0.9882
Vehicle
HFG
HHT

Fig. S11

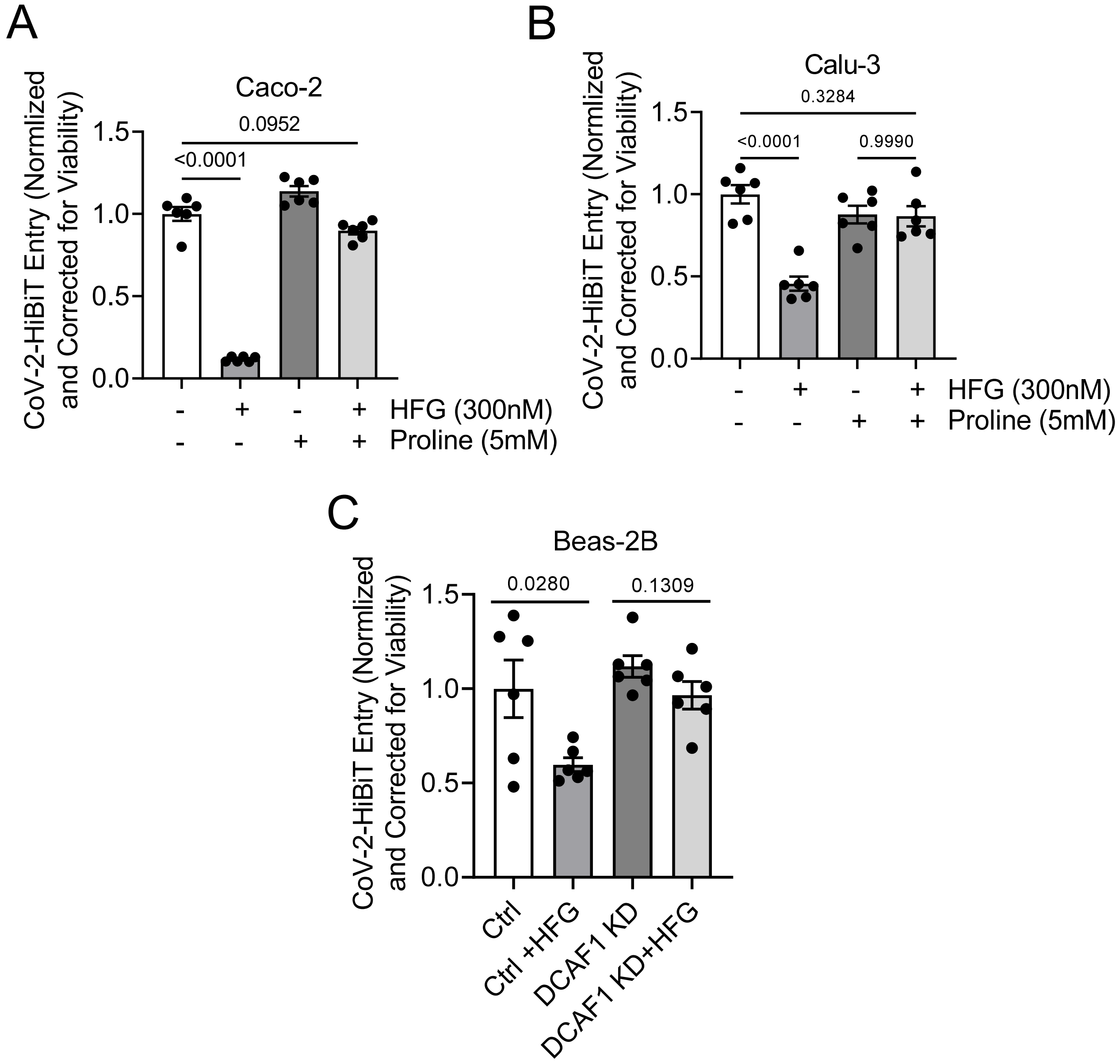

A
Caco-2
CoV-2-HiBiT Entry (Normlized and Corrected for Viability)
1.5
1.0
0.5
0.0
0.0952
<0.0001
- + - + HFG (300nM)
- - + + Proline (5mM)
B
Calu-3
CoV-2-HiBiT Entry (Normlized and Corrected for Viability)
0.3284
<0.0001
0.9990
1.5
1.0
0.5
0.0
- + - + HFG (300nM)
- - + + Proline (5mM)
C
Beas-2B
CoV-2-HiBiT Entry (Normlized and Corrected for Viability)
0.0280
0.1309
1.5
1.0
0.5
0.0
Ctrl
Ctrl +HFG
DCAF1 KD
DCAF1 KD+HFG

Fig. S12

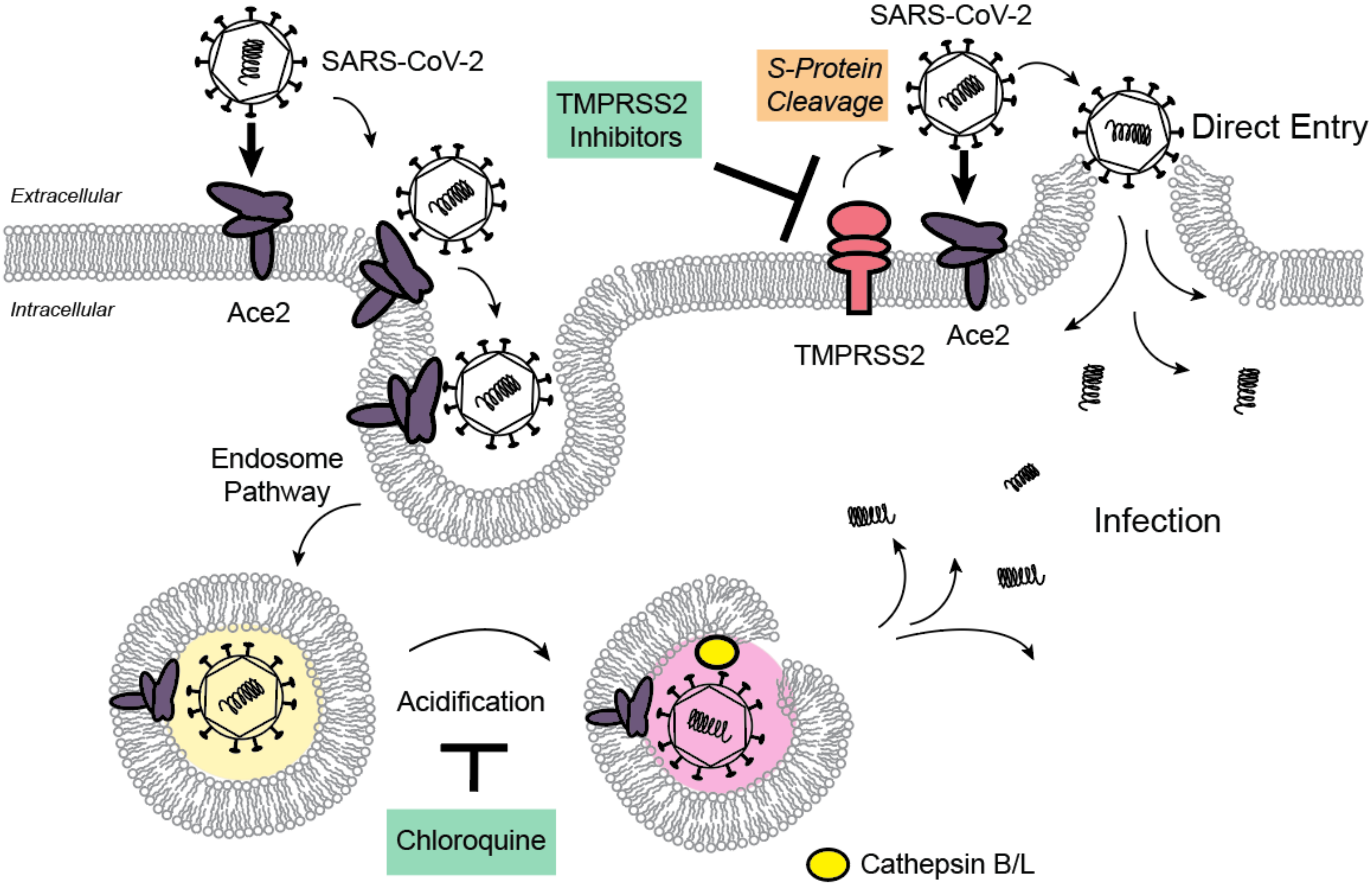

SARS-CoV-2
Extracellular
Intracellular
Ace2
Endosome
Pathway
TMPRSS2
Inhibitors
S-Protein
Cleavage
SARS-CoV-2
Direct Entry
TMPRSS2
Ace2
Infection
Acidification
Chloroquine
Cathepsin B/L
