# Supplementary material for: A high-throughput screen for TMPRSS2 expression identifies FDA-approved compounds that can limit SARS-CoV-2 entry
Source: Nat Commun. 2021 Jun 23;12:3907. doi: 10.1038/s41467-021-24156-y (PMC8222394; doi:10.1038/s41467-021-24156-y)
Supplement: Supplementary file 3 — Reporting Summary [file 41467_2021_24156_MOESM3_ESM.pdf]

nature research

Corresponding author(s): Chen BB

Last updated by author(s): May 27, 2021

Reporting Summary

Nature Research wishes to improve the reproducibility of the work that we publish. This form provides structure for consistency and transparency in reporting. For further information on Nature Research policies, see our Editorial Policies and the Editorial Policy Checklist.

Statistics

For all statistical analyses, confirm that the following items are present in the figure legend, table legend, main text, or Methods section.

n/a	Confirmed	
☐	☒	The exact sample size (*n*) for each experimental group/condition, given as a discrete number and unit of measurement
☐	☒	A statement on whether measurements were taken from distinct samples or whether the same sample was measured repeatedly
☐	☐	The statistical test(s) used AND whether they are one- or two-sided<br>*Only common tests should be described solely by name; describe more complex techniques in the Methods section.*
☒	☐	A description of all covariates tested
☒	☐	A description of any assumptions or corrections, such as tests of normality and adjustment for multiple comparisons
☐	☒	A full description of the statistical parameters including central tendency (e.g. means) or other basic estimates (e.g. regression coefficient) AND variation (e.g. standard deviation) or associated estimates of uncertainty (e.g. confidence intervals)
☐	☒	For null hypothesis testing, the test statistic (e.g. *F*, *t*, *r*) with confidence intervals, effect sizes, degrees of freedom and *P* value noted<br>*Give P values as exact values whenever suitable.*
☒	☐	For Bayesian analysis, information on the choice of priors and Markov chain Monte Carlo settings
☒	☐	For hierarchical and complex designs, identification of the appropriate level for tests and full reporting of outcomes
☒	☐	Estimates of effect sizes (e.g. Cohen's *d*, Pearson's *r*), indicating how they were calculated

Our web collection on statistics for biologists contains articles on many of the points above.

Software and code

Policy information about availability of computer code

Data collection	HTS data were collected using BioTek Gen5 software with Cytation5 plate reader system
Data analysis	Agilent VWORKs (12.3.0.1346), Thermo Momentum (5.3, Build 20190624.12), GraphPad (PRISM) 9.0

For manuscripts utilizing custom algorithms or software that are central to the research but not yet described in published literature, software must be made available to editors and reviewers. We strongly encourage code deposition in a community repository (e.g. GitHub). See the Nature Research guidelines for submitting code & software for further information.

Data

Policy information about availability of data

All manuscripts must include a data availability statement. This statement should provide the following information, where applicable:

- Accession codes, unique identifiers, or web links for publicly available datasets
- A list of figures that have associated raw data
- A description of any restrictions on data availability

The datasets generated during and/or analyzed during the current study are contained in this manuscript file or are available from the corresponding author upon reasonable request.

Field-specific reporting

Life sciences study design

All studies must disclose on these points even when the disclosure is negative.

Sample size	Sample size calculations were not conducted for in vitro experiments, however based on pilot immunoblotting studies, we determined that a sample size of n=3 independent experiments was considered adequate to detect an approximate 2-fold difference in protein abundance. For HTS studies, a Z factor was calculated and "hits" subsequently individually validated in complementary in vitro assays.
Data exclusions	No data were excluded from the analysis.
Replication	For HTS drug screening, "top-hits" were replicated and validated by subsequent IC50 experiments and in vitro experiments. At least n=3 biologically independent replicates were measured.<br>For in vitro data, experiments were conducted n=3 independent times. No data were excluded from the HTS or in vitro replication experiments.
Randomization	Randomization was not relevant for these studies, as we used stably expressing cell lines that were selected to generate monoclonal population for screening, which greatly reduced cellular heterogeneity that would have necessitated randomization into treatment groups.
Blinding	HTS experiments were automated and the identities of the individual compounds and esiRNA's were not known to the operator prior to performing the screen. For replication of in vitro findings, at least 1 independent experiment was carried out in a blinded fashion by two separate operators (1 operator treating cells in vitro unblinded and 1 operator processing immunoblots in a blinded fashion)

Reporting for specific materials, systems and methods

We require information from authors about some types of materials, experimental systems and methods used in many studies. Here, indicate whether each material, system or method listed is relevant to your study. If you are not sure if a list item applies to your research, read the appropriate section before selecting a response.

Materials & experimental systems

n/a	Involved in the study	
☐	☒	Antibodies
☐	☒	Eukaryotic cell lines
☒	☐	Palaeontology and archaeology
☒	☐	Animals and other organisms
☒	☐	Human research participants
☒	☐	Clinical data
☒	☐	Dual use research of concern

Methods

n/a	Involved in the study	
☒	☐	ChIP-seq
☒	☐	Flow cytometry
☒	☐	MRI-based neuroimaging

Antibodies

Antibodies used	Mouse monoclonal anti–V5 Tag (ThermoFisher, R960-25)<br>TMPRSS2 antibody (ThermoFisher, PA5-83286)<br>β-actin antibody (Invitrogen, MA5-15739)<br>HA antibody (Invitrogen, 2-2.2.14, #26183)<br>Ubiquitin antibody (Cell Signaling Technologies, 3933)<br>E-cadherin antibody (Santa Cruz Biotechnologies, G-10, sc-8426)<br>GFP antibody (Cell Signaling Technologies, 4B10, 2955)<br>Dilutions are noted in the manuscript methods section.
Validation	HA, GFP, and V5 antibodies was verified by relative expression in cells of a tagged-plasmid both by the manufacturer and in house. TMPRSS2 antibody was validated through ectopic expression of TMPRSS2 plasmid and RNAi validation in house. All antibodies were also validated by inspection of signal at the expected target size. Uncropped gel images are included with the manuscript source data.

Eukaryotic cell lines

Policy information about cell lines

Cell line source(s)	Beas-2b, MLE-12, Caco-2, HEK293T, Calu-3, and Vero cells were from ATCC.
Authentication	Cell lines were authenticated by ATCC and used within 20 passages from thawing commerical samples.
Mycoplasma contamination	All cell lines were tested negative for mycoplasma contamination

Commonly misidentified lines<br>(See ICLAC register)	No commonly misidentified cell lines were used in this study
