# Supplementary material for: A high-throughput screen for TMPRSS2 expression identifies FDA-approved compounds that can limit SARS-CoV-2 entry
Source: Nat Commun. 2021 Jun 23;12:3907. doi: 10.1038/s41467-021-24156-y (PMC8222394; doi:10.1038/s41467-021-24156-y)
Supplement: Supplementary file 4 — Source Data [file 41467_2021_24156_MOESM4_ESM.zip › Source Data/Uncropped Blots.pdf]

Fig.4A

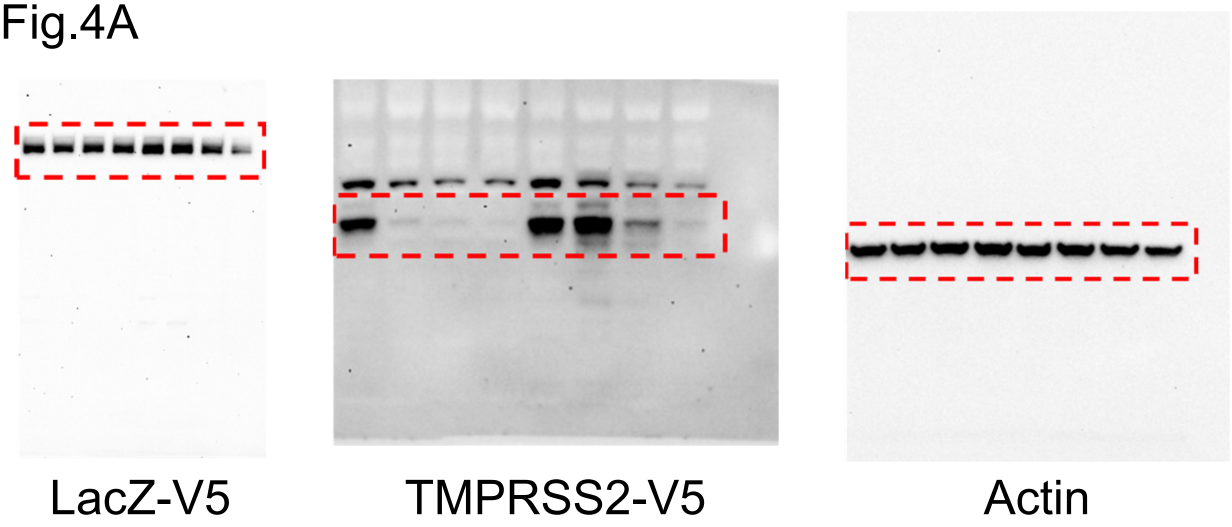

Fig.4B

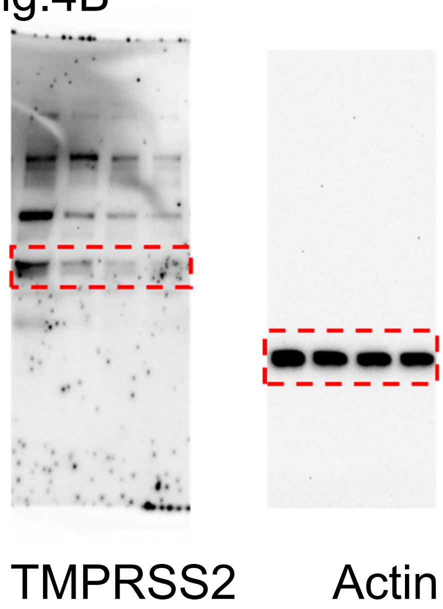

Fig.4C

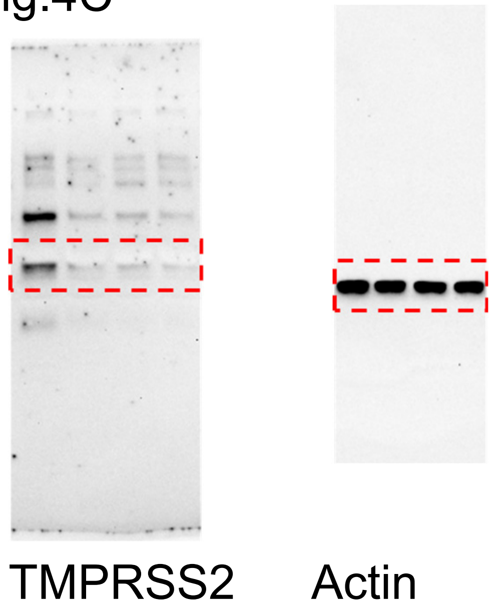

Fig.4D

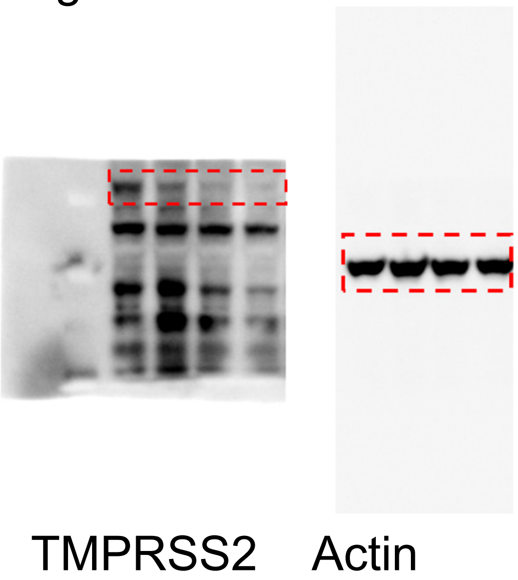

Fig.4E

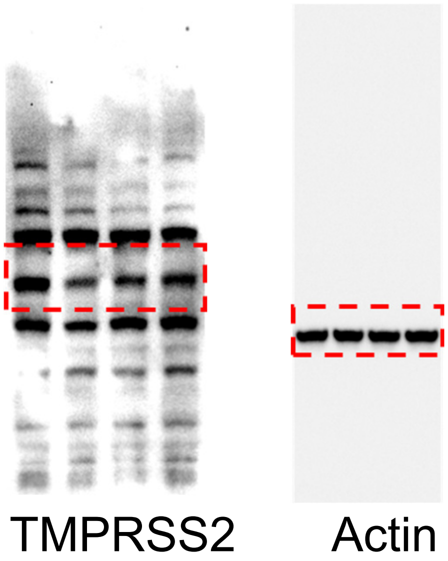

Fig.5A

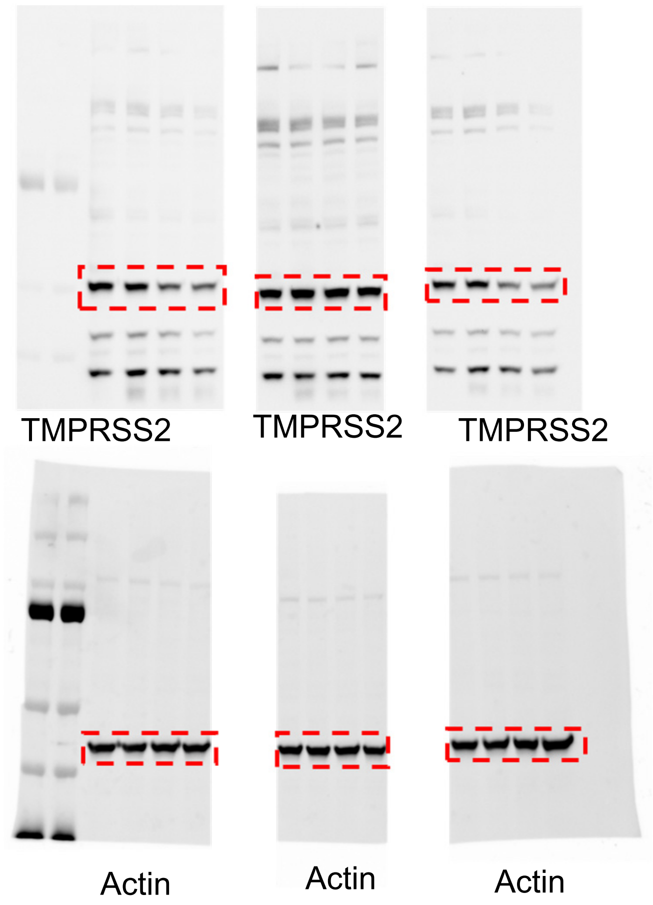

Fig.5D

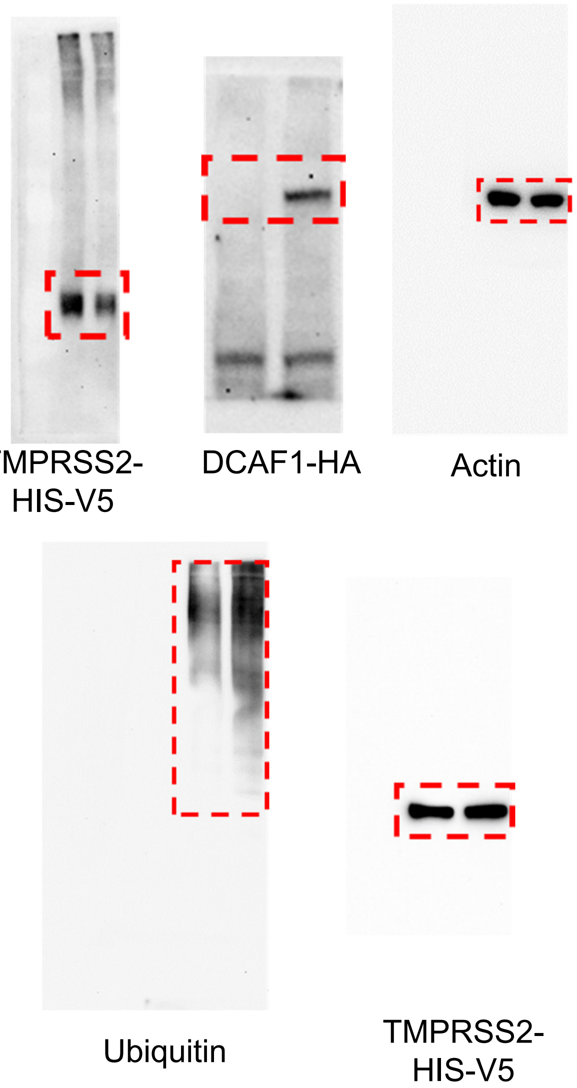

Fig.5E

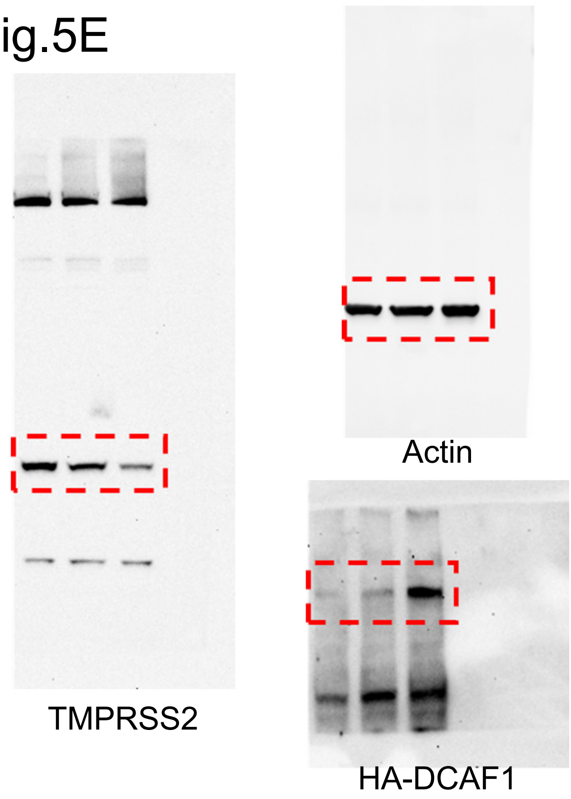

Fig.5F

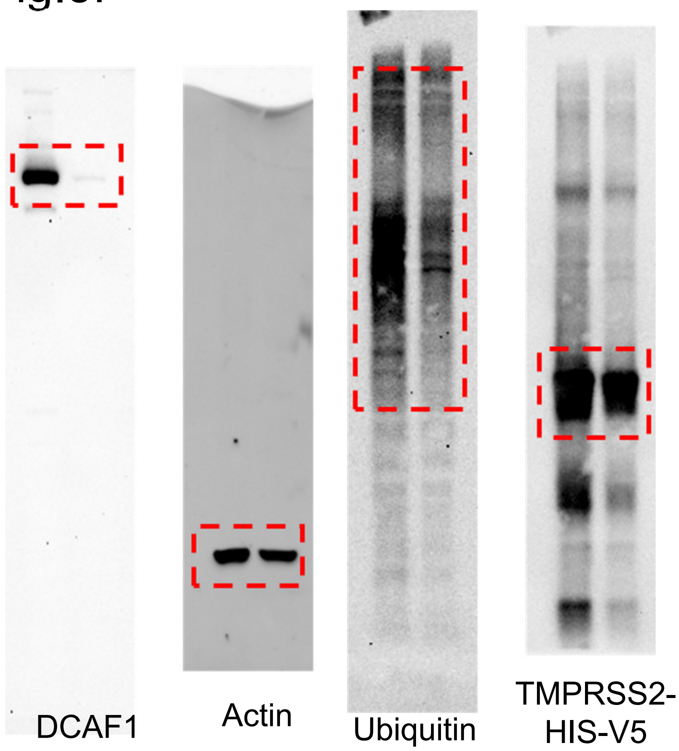

Fig.6A

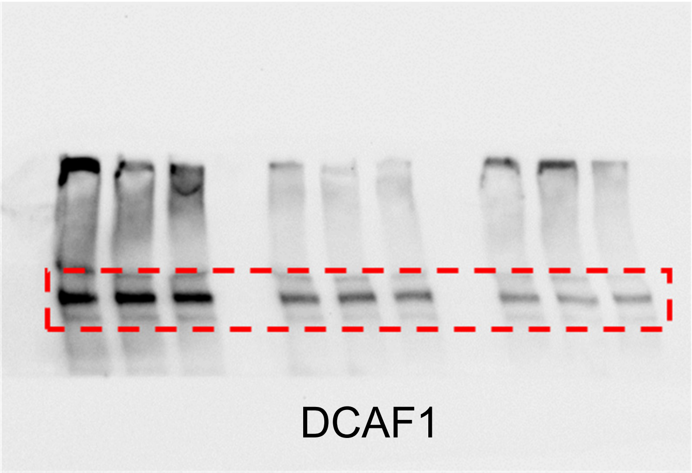

DCAF1

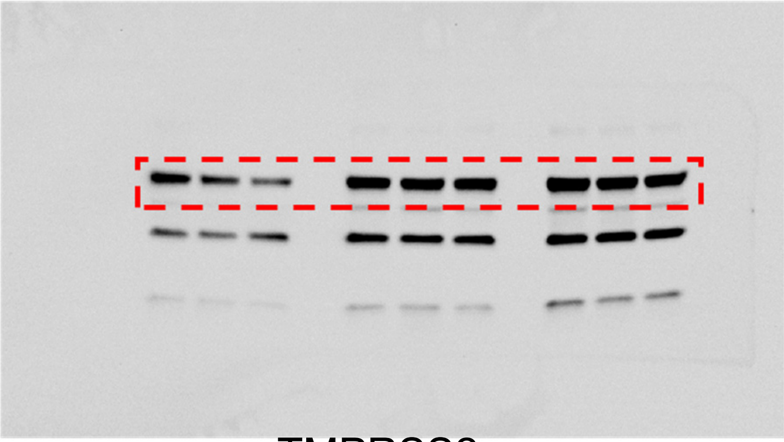

TMPRSS2

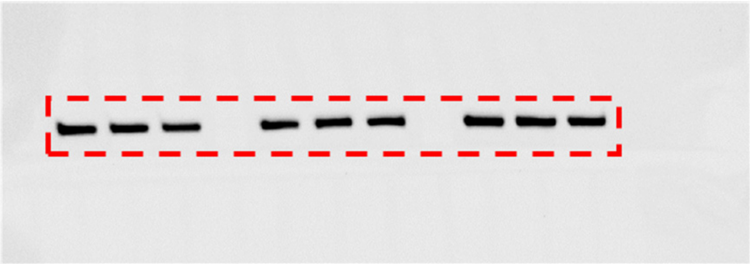

E-cadherin

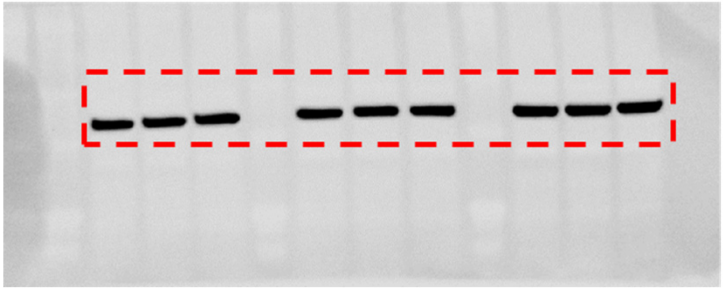

Actin

Fig.6C

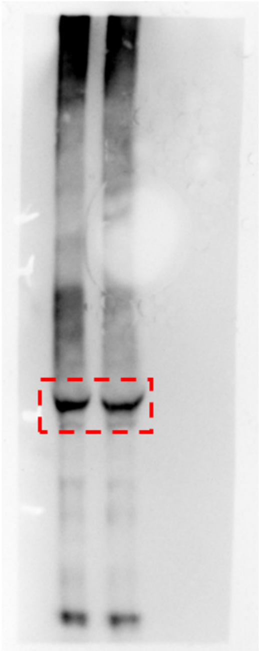

TMPRSS2
(HiBiT Blotting)

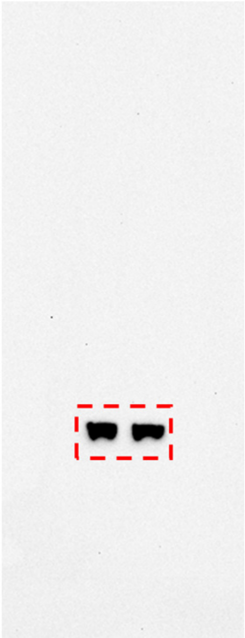

Actin

Fig.7H

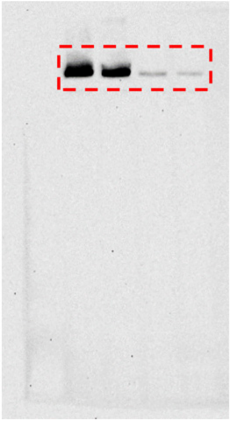

DCAF1

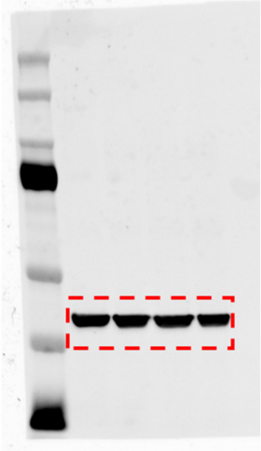

Actin

Fig.S3A

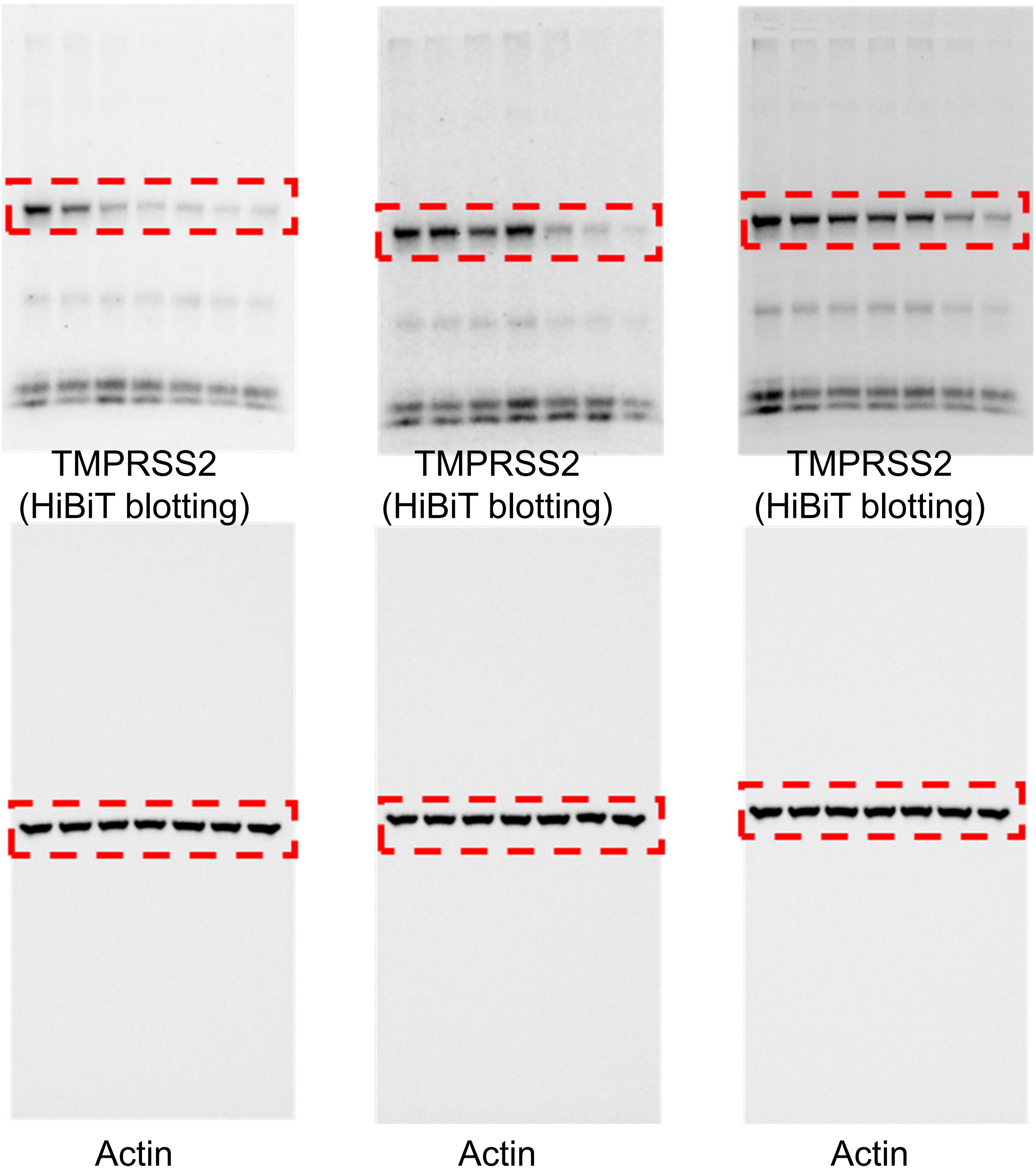

TMPRSS2
(HiBiT blotting)
TMPRSS2
(HiBiT blotting)
TMPRSS2
(HiBiT blotting)
Actin
Actin
Actin

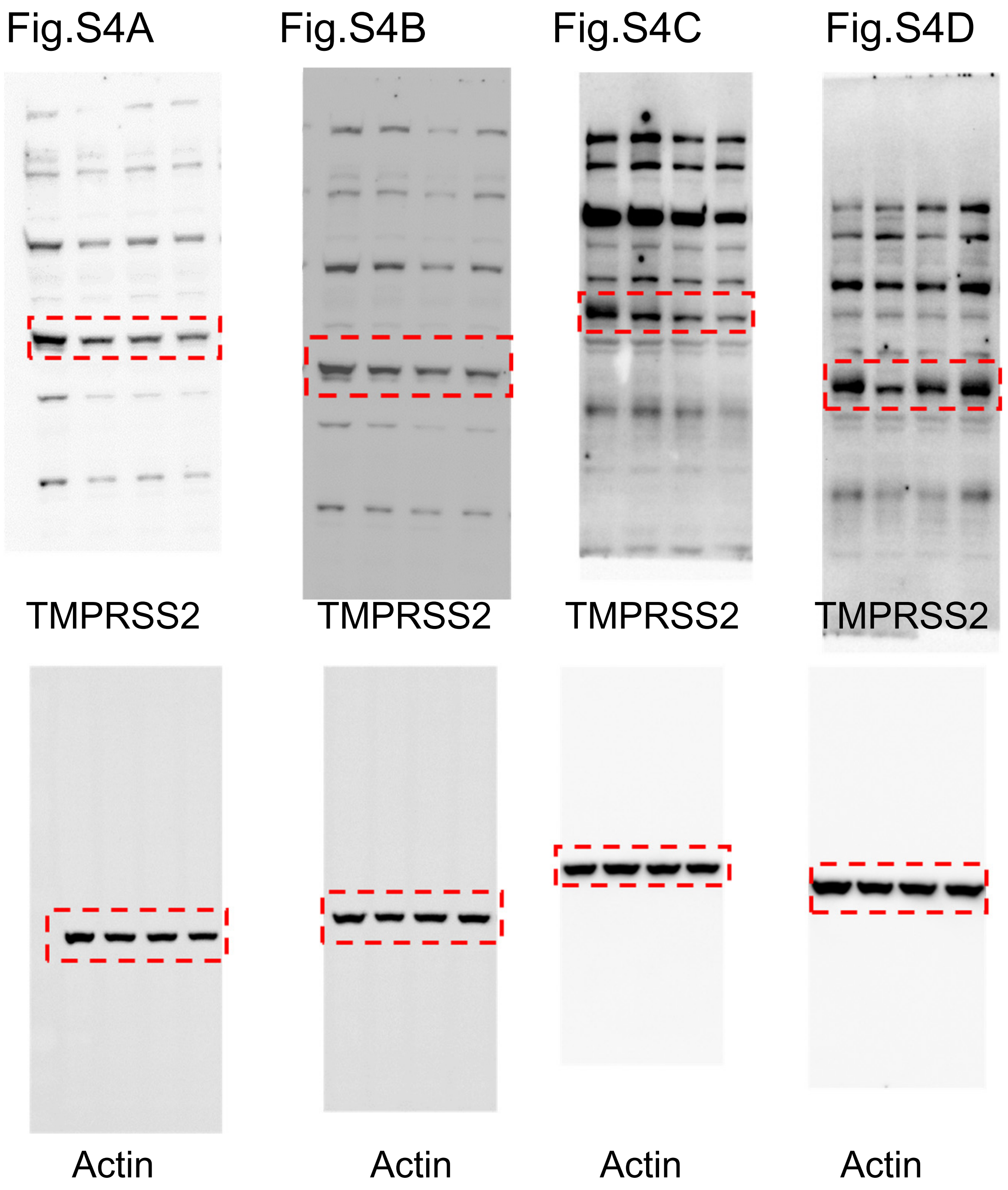
Fig.S4A
Fig.S4B
Fig.S4C
Fig.S4D
TMPRSS2
TMPRSS2
TMPRSS2
TMPRSS2
Actin
Actin
Actin
Actin

Fig.S5A

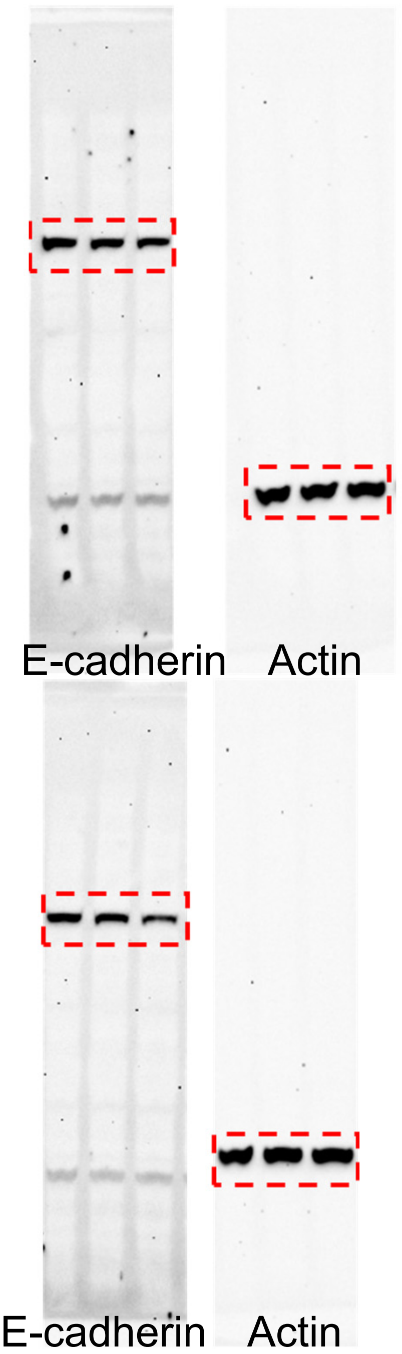

Fig.S5B

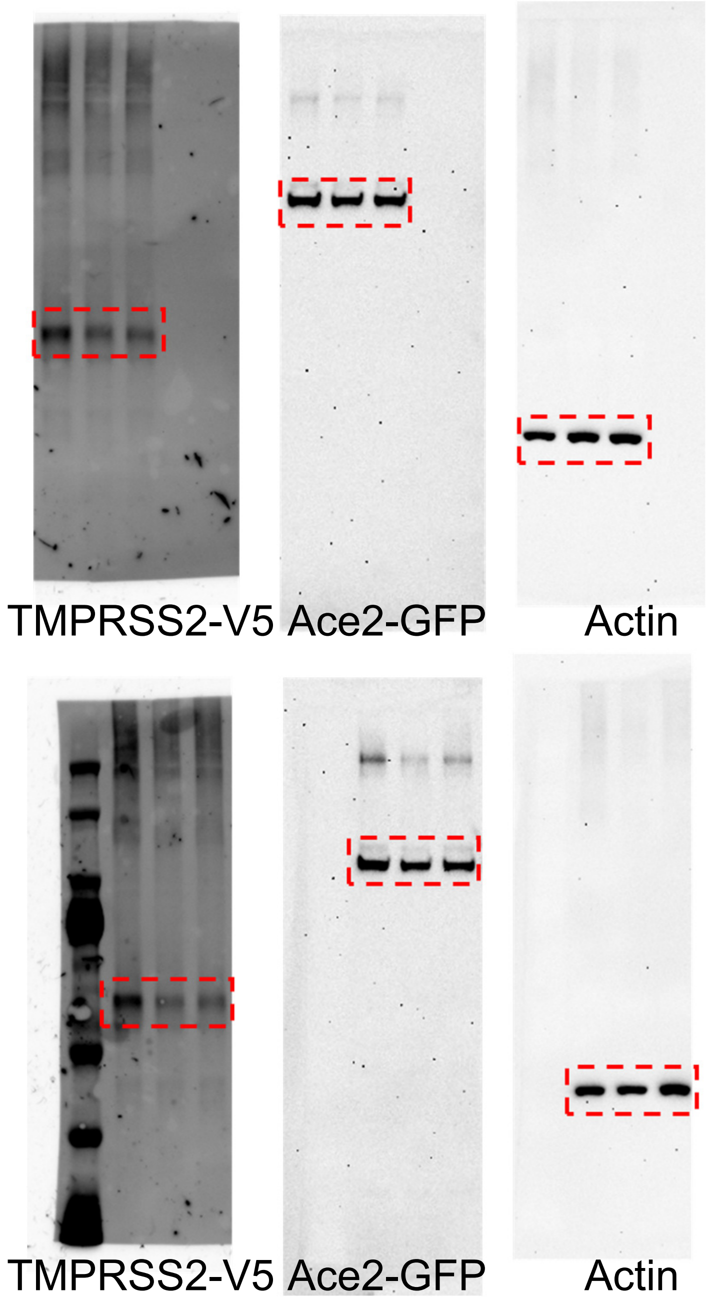

Fig.S5C

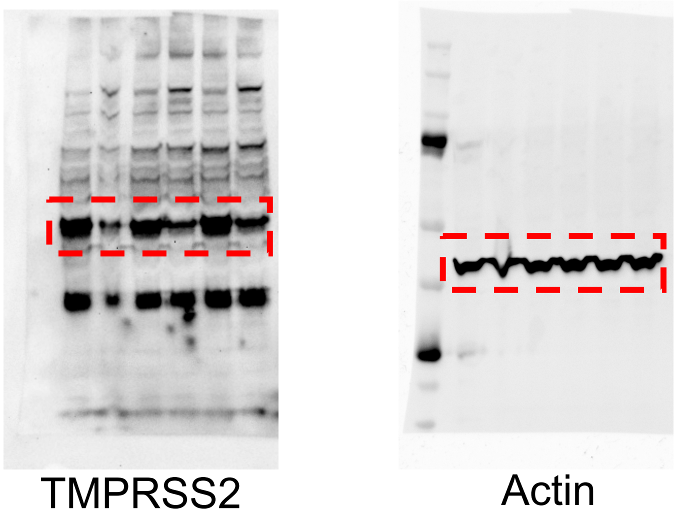

Fig.S6A

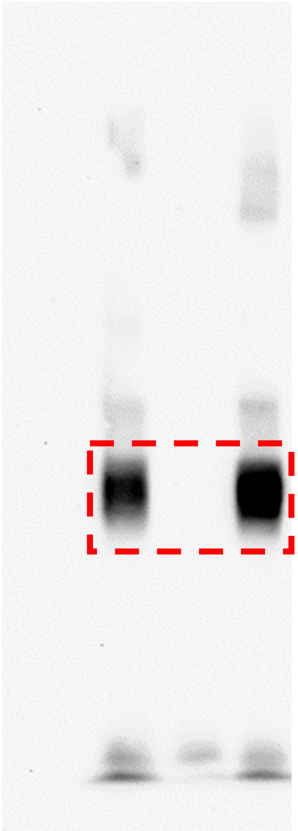

TMPRSS2-V5

Fig.S10A

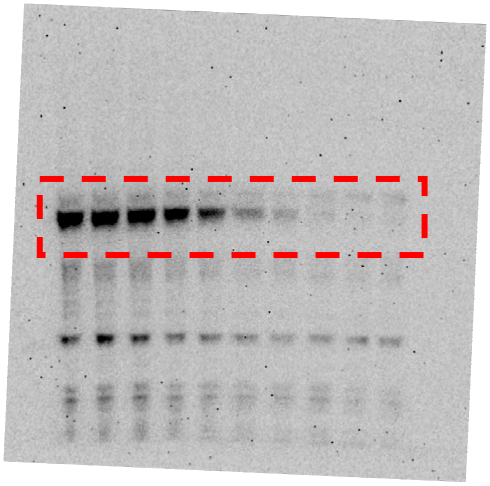

TMPRSS2
(HiBiT Blotting)

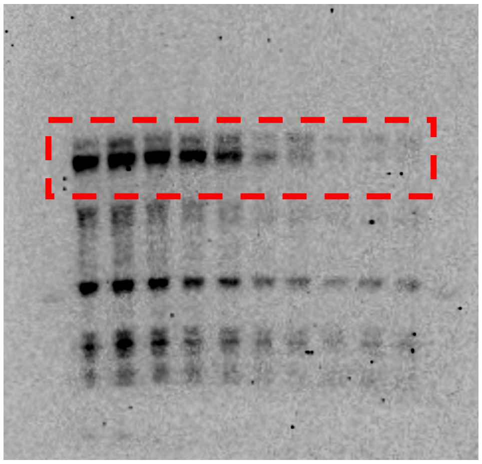

TMPRSS2
(HiBiT Blotting)

Fig.S10B

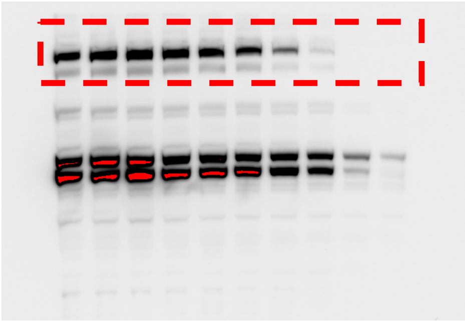

DCAF1

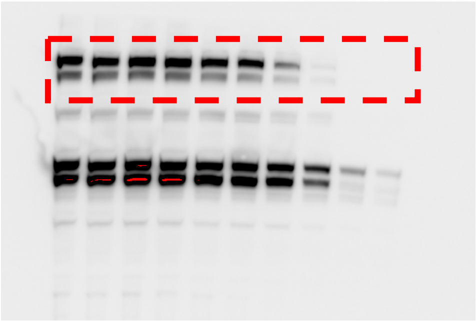

DCAF1
